# Long noncoding RNA *NONHSAT160169.1* promotes resistance via *hsa-let-7c-3p/*SOX2 axis in gastric cancer

**DOI:** 10.1038/s41598-023-47961-5

**Published:** 2023-11-27

**Authors:** Xuan Zhao, Zijian Xu, Bi Meng, Tong Ren, Xu Wang, Rui Hou, Sijin Li, Wen Ma, Dan Liu, Junnian Zheng, Ming Shi

**Affiliations:** 1 Cancer Institute, Xuzhou Medical University, 209 Tongshan Road, Xuzhou, 221004 Jiangsu China; 2 Center of Clinical Oncology, The Affiliated Hospital of Xuzhou Medical University, 99 Huaihai Road, Xuzhou, 221002 Jiangsu China; 3 Jiangsu Center for the Collaboration and Innovation of Cancer Biotherapy, Xuzhou Medical University, 209 Tongshan Road, Xuzhou, 221004 Jiangsu China; 4 College of Pharmacy, Xuzhou Medical University, 209 Tongshan Road, Xuzhou, 221004 Jiangsu China

**Keywords:** Cancer, Cancer genetics, Cancer therapy, Tumour biomarkers

## Abstract

In clinical trials involving patients with HER2 (ERBB2 receptor tyrosine kinase 2) positive gastric cancer, the efficacy of the HER2-targeted drug lapatinib has proven to be disappointingly poor. Under the persistent pressure exerted by targeted drug therapy, a subset of tumor cells exhibit acquired drug resistance through the activation of novel survival signaling cascades, alongside the proliferation of tumor cells that previously harbored mutations conferring resistance to the drug. This study was undertaken with the aim of elucidating in comprehensive detail the intricate mechanisms behind adaptive resistance and identifying novel therapeutic targets that hold promise in the development of effective lapatinib-based therapies for the specific subset of patients afflicted with gastric cancer. We have successfully established a gastric cancer cell line with acquired lapatinib resistance, designated as HGC-27-LR cells. Utilizing comprehensive coding and noncoding transcriptome sequencing analysis, we have identified key factors that regulate lapatinib resistance in HGC-27 cells. We have compellingly validated that among all the lncRNAs identified in HGC-27-LR cells, a novel lncRNA (long noncoding RNA) named *NONHSAT160169.1* was found to be most notably upregulated following exposure to lapatinib treatment. The upregulation of *NONHSAT160169.1* significantly augmented the migratory, invasive, and stemness capabilities of HGC-27-LR cells. Furthermore, we have delved into the mechanism by which *NONHSAT160169.1* regulates lapatinib resistance. The findings have revealed that *NONHSAT160169.1*, which is induced by the p-STAT3 (signal transducer and activator of transcription 3) nuclear transport pathway, functions as a decoy that competitively interacts with *hsa-let-7c-3p* and thereby abrogates the inhibitory effect of *hsa-let-7c-3p* on SOX2 (SRY-box transcription factor 2) expression. Hence, our study has unveiled the *NONHSAT160169.1*/*hsa-let-7c-3p*/SOX2 signaling pathway as a novel and pivotal axis for comprehending and surmounting lapatinib resistance in the treatment of HER2-positive gastric cancer.

## Introduction

Gastric cancer exhibits approximately 20% amplification of the ERBB2 receptor tyrosine kinase 2 (HER2) gene and overexpression of the HER2 protein^1^. Multiple novel drugs targeting HER2 drugs are currently being developed for gastric cancer, such as small molecular kinase inhibitors, monoclonal antibodies, antibody–drug conjugates, and other novel therapeutics^2^. One of the classical HER2-targeted kinase inhibitors is lapatinib; it exhibits the advantages of oral administration and favorable patient compliance, and is currently undergoing clinical trials for the treatment of gastric cancer^3^. Lapatinib is a dual inhibitor that competitively binds to the ATP-binding site of the EGFR/HER2 heterodimer, thereby blocking EGFR and HER2 tyrosine kinase activity and inhibiting cell proliferation^4^.

While molecular-targeted therapy holds promise as a therapeutic strategy for enhancing the overall survival (OS) of select patients, drug resistance poses a significant obstacle and remains a limiting factor in treatment outcomes^5^. Currently, patients with HER2-positive gastric cancer who exhibit persistent activation of mitotic signaling pathways are unresponsive to lapatinib treatment; this phenomenon is referred to as primary resistance^6^. However, the possibility of secondary drug resistance in drug-responsive patients remains a concern, as it may result in a decrease or loss of treatment effectiveness within the optimal time frame, which is brief^7^. Under prolonged exposure to targeted drug therapy, some tumor cells may develop adaptive drug resistance by altering their survival signals, in conjunction with the proliferation of cancer cells that originally possessed mutations contributing to drug resistance^8^.

The association between long non-coding RNAs and drug resistance in tumor-targeted therapy is currently a hot topic of research^9–11^. LncRNAs are aberrantly expressed at different stages of gastric cancer progression, and a few studies have reported mechanisms by which lncRNAs regulate lapatinib-induced drug resistance in gastric cancer; hence, lncRNAs associated with drug resistance may potentially be targeted for personalized therapeutic strategies^11–13^. In this study, through transcriptome microarray, we aimed to identify novel lncRNAs associated with lapatinib resistance as well as key genes that interact with lncRNAs in gastric cancer to establish a signal regulatory network. Our findings may aid in predicting drug sensitivity in patients with gastric cancer and devising tailored treatment strategies for individual patients.

## Materials and methods

### Cell culture

Gastric cancer cell lines, namely HGC-27 (TCHu 22) and NCI-N87 (SCSP-534), were procured from the Cell Bank of the Chinese Academy of Sciences (Shanghai, China). Lapatinib-resistant HGC-27 (HGC-27-LR) cells were established using the classical method of drug gradient pressure in vitro. HGC-27 and HGC-27-LR cells were cultured in high glucose-DMEM (D5796; Sigma) supplemented with 10% fetal bovine serum (FBS) (P30922; TransGen Biotech). All cells were cultured at 37 °C in a humidified incubator with 5% CO_2_.

### Cell counting kit-8 (CCK-8) assay

HGC-27, HGC-27-LR, LV-vector-HGC-27, LV-*NONHSAT160169.1*-HGC-27, shRNA-NC-HGC-27-LR, or shRNA-*NONHSAT160169.1*-HGC-27-LR cells were seeded in 96-well plates (6 × 10^3^ cells per well) and cultured overnight. The CCK-8 reaction reagent was diluted (1:10) in cell culture medium. Following the treatment of cells with lapatinib, the old medium in the 96-well plate was discarded, and 100 μL of diluted CCK-8 reaction reagent was added to each well. The plates were then incubated at 37 °C with 5% CO_2_ for 2 h. Absorbance at 450 nm was measured using a microplate reader (Bio-Rad). Relative cell viability was calculated as a percentage of the control group.

### Scratch wound healing assay

HGC-27 or HGC-27-LR cells were seeded in six-well plates and cultured until they were 100% confluent. Scratch wounds were created by dragging a 200 μL pipette tip into cell monolayers. Before replacing the conditioned medium, cells were washed three times with 1 × PBS (0.1 M PBS, pH 7.4) to eliminate non-adherent cellular debris. Cell migration to the wound surface was monitored and subsequently measured for distance.

### Transwell assay

In brief, 2 × 10^5^ cells were seeded into the upper chambers of a transwell (TCS-013-024, 8.0 μm; BIOFIL) and suspended in serum-free medium overnight. The upper compartment of transwell filter inserts contained serum-free culture medium, while the lower compartment contained culture medium supplemented with 15% serum with or without lapatinib. Following incubation at 37 °C and 5% CO_2_, cells were fixed with paraformaldehyde for 30 min, washed three times with 1 × PBS, stained with crystal violet (0.1%) for 30 min, washed with 1 × PBS to remove the excess dye, dried at room temperature, and then photographed.

### Sphere formation assay

HGC-27, HGC-27-LR, LV-vector-HGC-27, LV-*NONHSAT160169.1*-HGC-27, shRNA-NC-HGC-27-LR, shRNA-*NONHSAT160169.1*-HGC-27-LR, and LV-SOX2-HGC-27 cells were respectively seeded in 6-well (2000 cells/well) and 24-well (1000 cells/well) plates (ultra-low adsorption; Corning) after being suspended as single cells. DMEM/F12 supplemented with 10% FBS, penicillin–streptomycin-amphotericin B solution (100 U/mL), l-glutamine (2 mM), EGF (human, 20 ng/mL), B27 (1 ×), and bFGF (human, 20 ng/mL) were used as culture medium. Every two days, the culture medium was changed. After four or seven days, the spherical state of the cells was observed and photographed using a fluorescence microscope (Olympus). The number of spheres in the entire field of view was counted.

### Protein preparation of cells and western blot experiments

The cells were lysed using RIPA lysis buffer (C1053; APPLYGEN) containing cocktail (P8340; Sigma) and PMSF (0.1 mM). Subcellular localization of p-STAT3 in HGC-27 and HGC-27-LR cells was determined using a nuclear and cytoplasmic protein extraction kit (P0027; Beyotime). Cell lysates were centrifuged at 12,000 × *g*, 4 °C for 20 min, and the resulting supernatant was collected. The protein concentration in the supernatant was measured with a bicinchoninic acid (BCA) protein assay kit (P1511; APPLYGEN). Following 12% or 15% SDS-PAGE, proteins were transferred to a nitrocellulose filter (NC) membrane (NT-66485; Pall, BioTrace) using a semi-dry electrophoretic transfer system (Bio-rad). The membrane was blocked with 5% non-fat milk for 1 h at room temperature before being incubated with primary antibodies (1:1000) against CD133 (18470-1-AP; Proteintech), ALDH1A1 (sc-374149; Santa Cruz), ACTB (A-5441; Sigma), SOX2 (66411-1-Ig; Proteintech), p-STAT3 (9145S; Cell Signaling Technology), STAT3 (4904S; Cell Signaling Technology), GAPDH (60004-1-Ig; Proteintech), and histone H3 (4499P; Cell Signaling Technology) at 4 °C overnight. The cells were then washed three times with 1 × TBST (1 × TBS + 0.1% TWEEN 20), and then incubated with secondary antibodies (goat anti-rabbit, 7074P2; goat anti-mouse, 7076P2; Cell Signaling Technology) for 1 h at room temperature. After incubation with horseradish peroxidase (HRP) substrate for 3 min, fluorescence signals were detected using Tanon 5200 Multi. The relative levels of the proteins were quantified using Image J software.

### Quantitative real-time PCR (qPCR)

Total RNA was extracted using Trizol (9109; TAKARA). RNA was reverse-transcribed to cDNA using the PrimeScript RT Reagent Kit (DRR047; TAKARA). The reaction volume for qPCR was 20 μL comprising 10 μL of SYBR qPCR Master Mix (Q321-02; Vazyme), forward primers, reverse primers, template cDNA, and nuclease-free H_2_O. The relative mRNA level of a specific gene was normalized to that of beta-actin. The primer sequences are provided in the supplementary material (Table S1).

### RNA-fluorescent in situ hybridization (RNA-FISH)

*NONHSAT160169.1* probes with 5′ CY3 labeling were synthesized by Invitrogen. HGC-27 and HGC-27-LR cells were fixed in 4% paraformaldehyde and washed three times with 1 × PBS. Next, the cells were permeabilized using 0.1% Triton X-100. The cells were then washed three times with 1 × PBST (1 × PBS + 0.1% TWEEN 20), pre-hybridized with hybridization solution, and then incubated with 5′ CY3-labeled *NONHSAT160169.1* probe overnight. 4′, 6-diamidino-2-phenylindole (DAPI; D8417; Sigma-Aldrich) was used to indicate cell nuclei. Samples were observed and photographed using a confocal fluorescence microscope (LSM880; Zeiss, Germany).

### Construction of stable knockdown or overexpression cells

Lentivirus packaging of vector, *NONHSAT160169.1*, shRNA-NC, shRNA-*NONHSAT160169.1*, and *SOX2* were supplied by GeneChem Co., Ltd. (Shanghai, China). HGC-27 or HGC-27-LR cells were seeded in six-well plates (1 × 10^5^ cells per well) and cultured in high-glucose DMEM (D5796; Sigma) supplemented with 10% FBS. The plasmids and the infection reagent (HiTransG P) were thoroughly mixed and added to the wells. Following incubation at 37 °C with 5% CO_2_ for 16 h, the medium was replaced with fresh medium. After 48–72 h, the transfection efficiency was determined. Then, stable cell lines were established by screening with puromycin (2 μg/mL).

### Transient transfection

Specific miRNA mimics, inhibitors, and scrambling shRNA (NC) were designed and synthesized for *hsa-let-7c-3p* by Invitrogen (Table S2). At 50% cell confluence, mimics or inhibitor (negative control: NC) were transfected using Lipofectamine 2000 (11668-019; Invitrogen) according to the instruction.

### Luciferase activity assay

The binding sites between *NONHSAT160169.1* (623–1122 bp) and *hsa-let-7c-3p* and the sequence of the mutations in the binding site were inserted into a luciferase reporter plasmid (GV272, SV40-firefly-Luciferase-MCS; GeneChem Co., Ltd.). HGC-27-LR cells were seeded into 24-well plates (2 × 10^5^ cells/well) and cultured overnight in DMEM-H medium and 10% FBS. When cells were 50% confluent, luciferase reporter plasmids Luc-*NONHSAT160169.1*-WT and Luc-*NONHSAT160169.1*-MUT were co-transfected for 24 h with *hsa-let-7c-3p* mimics or NC and pGMLR-TK luciferase reporter (Renilla luciferase) plasmid. According to the manufacturer’s instructions from Dual Luciferase Reporter Assay Kit (Vazyme; DL101-01), luciferase activity was analyzed. Firefly luciferase activity was normalized to that of Renilla luciferase.

### Chromatin immunoprecipitation (ChIP) assay

ChIP assay was performed using the BeyoChIP™ Chromatin Immunoprecipitation (ChIP) Assay Kit with Protein A/G Magnetic Beads (P2080S; Beyotime Biotechnology). Briefly, HGC-27-LR cells were treated with 1% formaldehyde at room temperature to cross-link target proteins and genomic DNA. Then, HGC-27-LR cells were lysed using an SDS lysis buffer containing protease inhibitors. Ultrasonic shearing of genomic DNA to 200–1000 bp was performed at 4 °C, and genomic DNA was co-incubated with antibodies against p-STAT3 (1 μg; 9145S; Cell Signaling Technology) at 4 °C overnight for ChIP. IgG and POLR2A (RNA polymerase II subunit A) (2629S; Cell Signaling Technology) were used as negative and positive controls, respectively. Next, 80 µL of Protein A/G Magnetic Beads/Salmon Sperm DNA were added to the system, which was then subjected to slow shaking at 4 °C for 1 h. The immunoprecipitated DNA was detected by qPCR.

### In vivo xenograft assay

Statement: All animal experiments were performed in accordance with the requirements of the American Veterinary Medical Association (AVMA) Animal Euthanasia Guidelines. NCG mice (female, 5 weeks; Gempharmatech Co., Ltd.) were randomly divided into four groups (n = 8 per group). The density of LV-vector-HGC-27, LV-*NONHSAT160169.1*-HGC-27, shRNA-NC-HGC-27-LR, or shRNA-*NONHSAT160169.1*-HGC-27-LR cells were adjusted to 7 × 10^6^ cells/100 μL PBS and then subcutaneously injected into each NCG mouse following anesthetization by isoflurane inhalation. At the end of this experiment, mice were placed in euthanasia cages, which were then filled with CO_2_ at a balanced rate to render them unconscious quickly and minimize their suffering as much as possible. During euthanasia, the breathing condition and eye color of each mouse were continuously observed. The mice were not taken out of the cage until they stopped breathing and lost their eye color. Tumor tissues were isolated and collected from each mouse. Xenograft tumors were analyzed to determine their volume and weight and then photographed. Tumor volume was calculated as follows: tumor volume = length × width^2^ × 0.52. All animal experiments were performed in accordance with the ARRIVE guidelines and approved by the Animal Ethics Experiment Committee of Xuzhou Medical University (202112A435).

### Immunohistochemistry (IHC)

Xenograft tumors were fixed in 4% paraformaldehyde, and then paraffin-embedded tissue sections were prepared. The tissue sections were deparaffinized and then boiled in citrate buffer (0.01 mol/L, pH 6.0) for 20 min to conduct antigen retrieval. Tissues were permeabilized with 0.3% Triton X-100 for 20 min at room temperature (not required for membrane proteins). The sections were then washed three times with 1 × PBST for 5 min, blocked with 10% goat serum for 1 h at room temperature, and then incubated with primary antibodies against Ki-67 (1:200; 12202S; Cell Signaling Technology) or CD31 (1:1200; 3528S; Cell Signaling Technology) overnight at 4 °C. The sections were then washed three times with 1 × PBST for 5 min, and the activity of endogenous HRP was blocked using 0.3% H_2_O_2_. The sections were then washed three times with 1 × PBST for 5 min, followed a incubation with secondary antibody for 1 h at 37 °C. The sections were washed three times with 1 × PBST for 5 min and reacted with diaminobenzidine (DAB) for 1–10 min. The sections were then washed with hematoxylin for 3 min and rinsed with distilled water. Next, the sections were subjected to dehydration, and the slices were sealed. Finally, sections were observed and photographed using a multispectral high-throughput IHC scanning system (Olympus, Japan).

### Statistical analysis

Experimental data are presented as means ± standard error of mean for at least three independent experiments. All statistical analyses were performed using SPSS 17.0 (SPSS Inc., Chicago, IL, USA) and GraphPad Prism 7 (GraphPad Software, San Diego, CA, USA). *P* < 0.05 was considered to indicate statistical significance.

### Ethics statement

All the animal experiments were performed in accordance with the ARRIVE guidelines and approved by the Animal Ethics Experiment Committee of Xuzhou Medical University (202112A435). At the end of the experiment, we euthanized the mice in strict accordance with the requirements of the American Veterinary Medical Association (AVMA) Animal Euthanasia Guidelines.

## Results

### Migration, invasion, and stem cell phenotypes were enhanced in lapatinib-resistant HER2-positive gastric cancer cells

**Figure 1:**
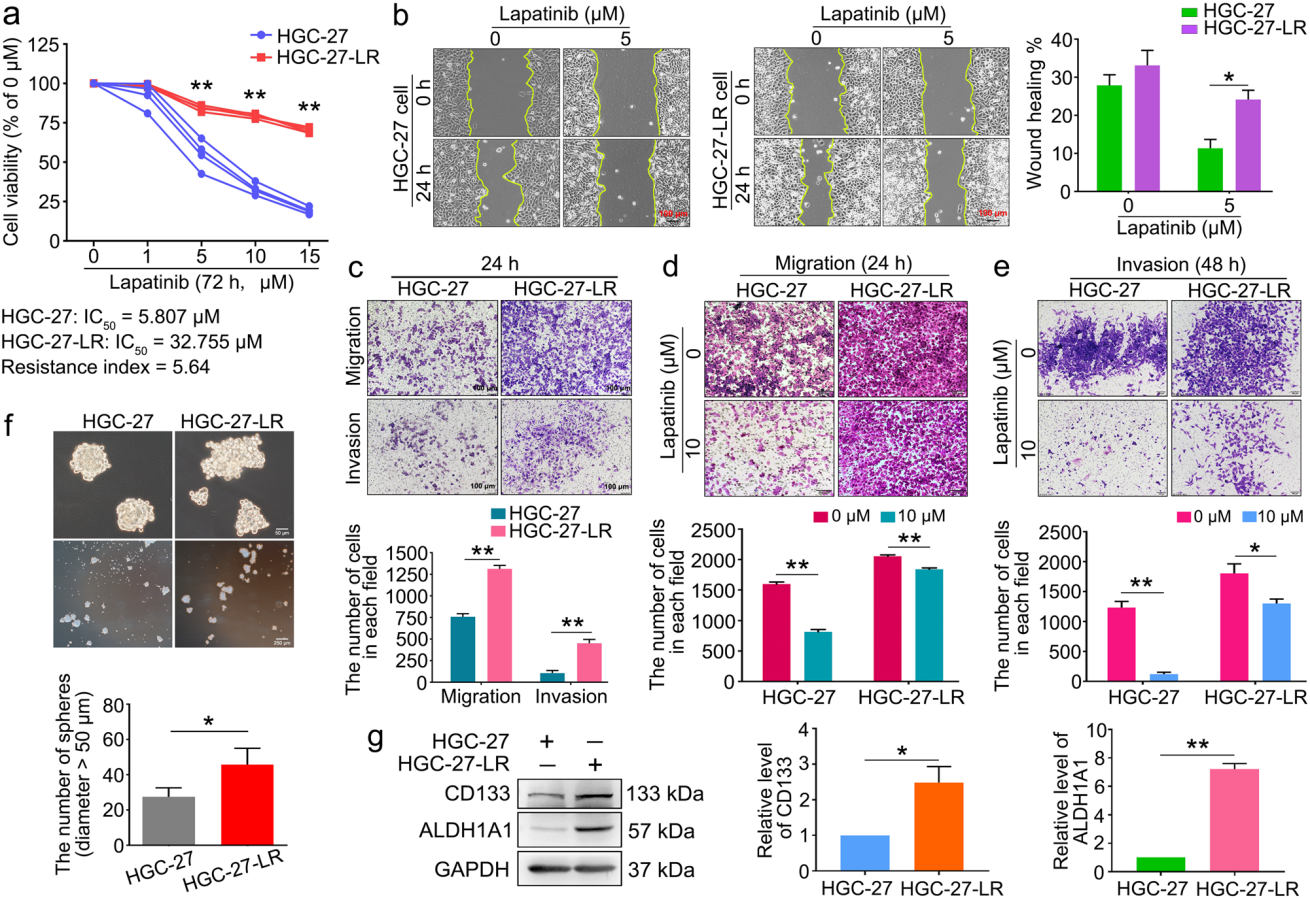
Migration, invasion, and stemness properties were enhanced in HGC-27-LR cells. (**a**) HGC-27 and HGC-27-LR cells were treated with various concentrations of lapatinib for 72 h. Cell viability was analyzed by CCK-8 assay, and then the IC_50_ value and drug resistance index of cells were calculated. (**b**) After making a scratch, we treated HGC-27 and HGC-27-LR cells with 5 μM lapatinib for 24 h. The wounds’ width was measured, and relative wound closure was quantified. (**c**) Transwell assays were performed to analyze migration and invasion of HGC-27 and HGC-27-LR cells, respectively. After 24 h, the cells were fixed and stained with crystal violet. Photographs were then captured and the cell counts were determined (scale bar = 100 μm). (**d**,**e**) HGC-27 and HGC-27-LR cells were treated with 10 μM lapatinib for 24 h or 48 h. Transwell assays were performed to analyze the migratory and invasive properties of the cells (Scale bar = 100 μm). (**f**) Sphere formation of HGC-27 and HGC-27-LR cells was analyzed after cultured in six-well ultra-low adhesion culture plates for 7 days. The number of spheres (diameter > 50 μm) was determined. (**g**) The protein levels of CD133 and ALDH1A1 in HGC-27 and HGC-27-LR cells were determined by western blot. Results are shown as mean ± SEM (*p < 0.05, **p < 0.01).

The HGC-27 cell line has been widely used in studies on gastric cancer progression, improvements in therapeutic strategies, and drug resistance^14–16^. HGC-27-LR cells were established using a classical approach to subjecting cells to long-term drug gradient pressure in vitro^9^. Subsequently, CCK-8 analysis was performed to determine any potential alterations in the sensitivity of HGC-27 cells to lapatinib. The results suggested that lapatinib inhibited the growth of HGC-27 cells in a concentration-dependent manner, while no such effect was observed in HGC-27-LR cells. The half-maximal inhibitory concentrations (IC_50_) of lapatinib for the inhibition of HGC-27 and HGC-27-LR were 5.807 μM and 32.755 μM, respectively. The resistance index (RI) was 5.64 (RI = IC_50_-HGC-27-LR cell/IC_50_-HGC-27 cell) (Fig. 1a). Compared to parental cells, the migration and invasive abilities of HGC-27-LR cells were significantly enhanced (Fig. 1b–e). Drug resistance due to alterations in the cell stemness phenotype during cancer treatment has been widely reported^17–19^. The sphere formation assay revealed that individual HGC-27-LR cells exhibited a greater capacity for self-renewal and cellular aggregation into clusters compared to individual HGC-27 cells (Fig. 1f). In addition, the levels of CD133 (prominin 1) and ALDH1A1 (aldehyde dehydrogenase 1 family member A1) proteins, which are markers for gastric tumor stem cells, were notably increased in HGC-27-LR cells (Fig. 1g). Altogether, these results indicated the successful establishment of a lapatinib-resistant cell line, which exhibited enhanced migration, invasion, and stemness phenotypes.

### Lapatinib-induced pressure triggered lncRNA NONHSAT160169.1 upregulation, which promoted migration, invasion, self-renewal abilities, and lapatinib resistance of HGC-27 cells

**Figure 2:**
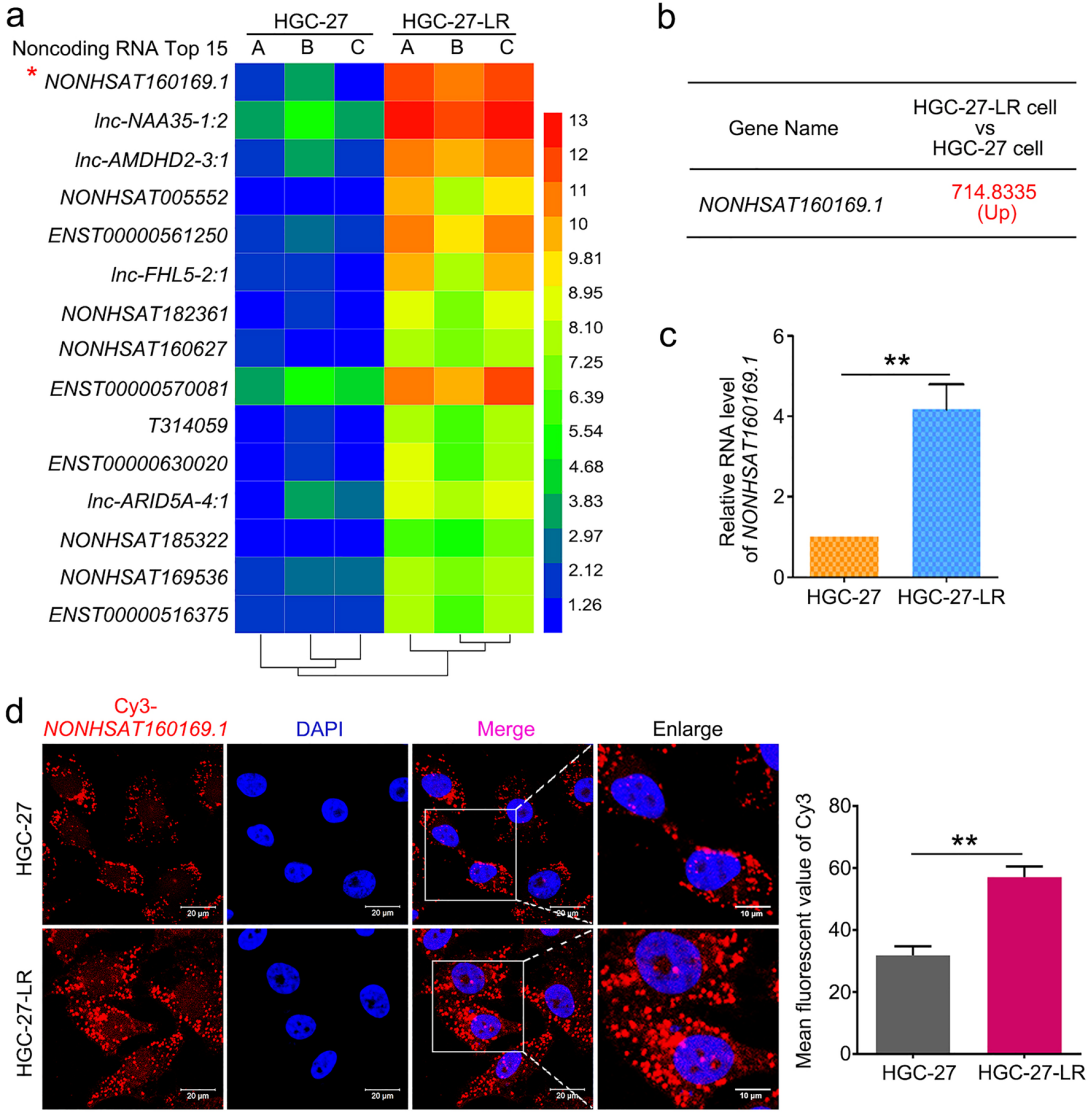
*NONHSAT160169.1* was significantly upregulated in HGC-27-LR cells. (**a**) A clustering heatmap of the top 15 upregulated non-coding RNAs in HGC-27-LR compared to those in HGC-27 cells. The heatmap image was generated using the Heml 1.0 software: http://hemi.biocuckoo.org/down.php. (**b**) The fold change in the upregulation of *NONHSAT160169.1*, as revealed by lncRNA microarray analysis. (**c**) RNA levels of *NONHSAT160169.1* in HGC-27 and HGC-27-LR cells were determined by qPCR. (**d**) RNA fluorescent in situ hybridization of *NONHSAT160169.1* (Scale bar = 10 μm, enlarged). Results are shown as mean ± SEM (**p < 0.01).

To identify novel noncoding RNAs and survival signaling mechanisms that contribute to lapatinib resistance in HGC-27 cells, we performed transcriptome microarray analysis for both coding and noncoding RNAs. Compared to HGC-27 cells, lncRNA *NONHSAT160169.1* in HGC-27-LR cells was most significantly increased in the microarray, with a fold change of 714.8 (Fig. 2a,b). *NONHSAT160169.1* was identified as a lncRNA using the NONCODE database (http://www.noncode.org/index.php). This lncRNA is located in the forward strand of chromosome 11 (hg38) and has a total length of 1384 bp (Table S3). However, despite a search of the NCBI database, no relevant information or research pertaining to *NONHSAT160169.1* was found. Therefore, this novel lncRNA needs to be investigated further. Initially, we analyzed the level of *NONHSAT160169.1* in both HGC-27 and HGC27-LR cells and its level under lapatinib treatment. The results indicated that *NONHSAT160169.1* was highly expressed in HGC-27-LR cells (Fig. 2c) (only the data of *NONHSAT160169.1*, which was closely associated with this study are shown; validation results of other candidate target genes are not shown). RNA-FISH analysis revealed that *NONHSAT160169.1* was distributed in both the nucleus and cytoplasm of HGC-27 and HGC-27-LR cells. Moreover, the fluorescence intensity of *NONHSAT160169.1* was markedly increased in HGC-27-LR cells (Fig. 2d).

### Overexpression of NONHSAT160169.1 promoted lapatinib resistance in HGC-27 cells

**Figure 3:**
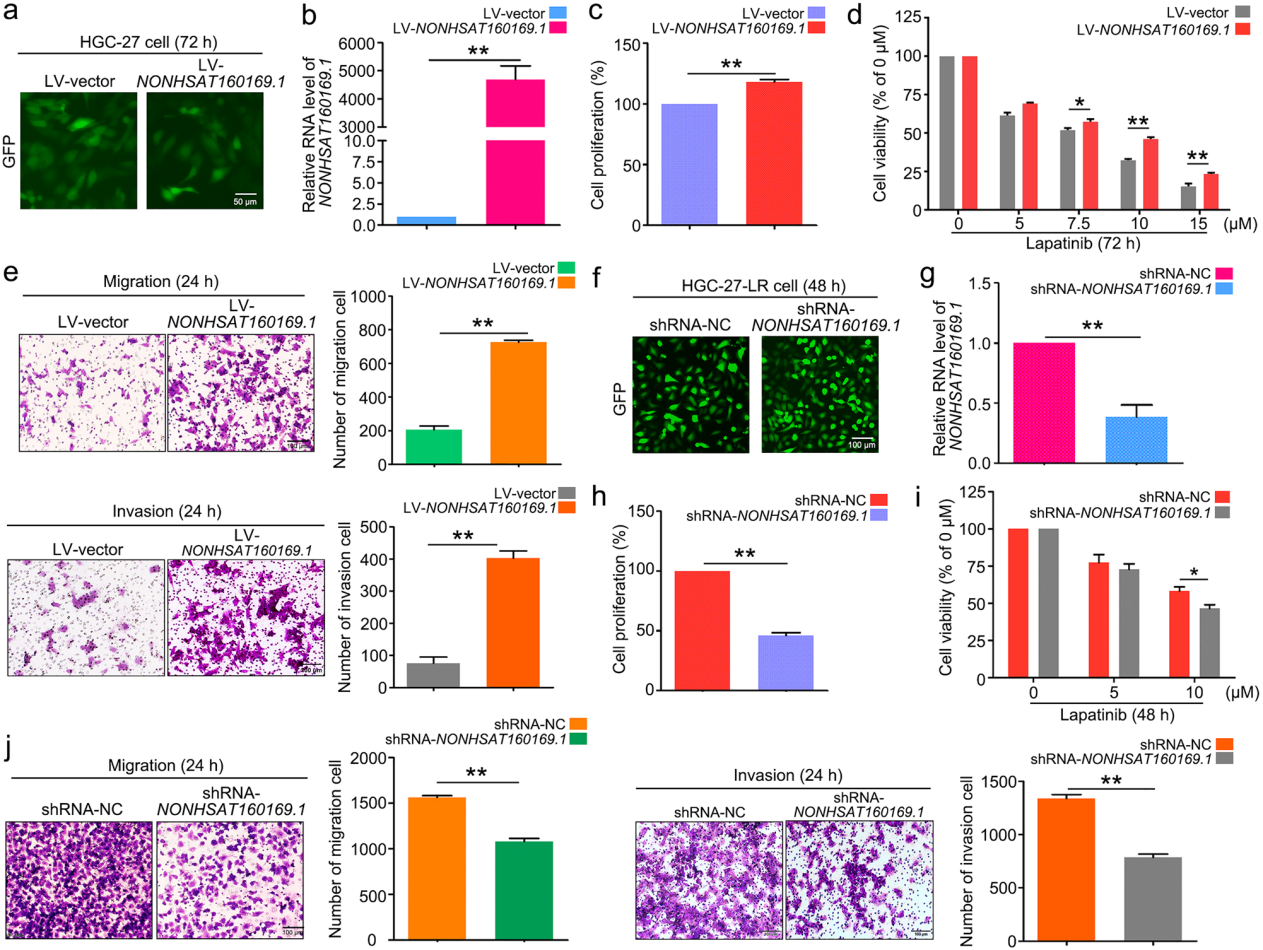
*NONHSAT160169.1* promoted lapatinib resistance, as well as migration and invasive properties of HGC-27 cells. (**a**) Fluorescence intensity of the GFP tag. (**b**) HGC-27 cells were infected with lentivirus that overexpressed *NONHSAT160169.1*, screened, and cultured with puromycin (2 μg/mL) for 1 week. qPCR analysis of the RNA level of *NONHSAT160169.1*. (**c**) CCK-8 analysis of of LV-vector and LV-*NONHSAT160169.1*-HGC-27 cell proliferation. (**d**) LV-vector and LV-*NONHSAT160169.1*-HGC-27 cells were treated with lapatinib for 72 h. Cell viability was analyzed by CCK-8 assay. (**e**) Transwell assays were performed to determine the migration and invasion of LV-vector and LV-NONHSAT160169.1-HGC-27 cells, respectively. After 24 h, the cells were fixed and stained with crystal violet. Photographs were captured and cell count was determined (Scale bar = 100 μm). (**f**) Fluorescence intensity of the GFP tag. (**g**) HGC-27-LR cells were infected with lentivirus to knockdown *NONHSAT160169.1*, screened, and cultured with puromycin (2 μg/mL) for 1 week. qPCR analysis of the RNA level of *NONHSAT160169.1*. (**h**) The proliferation of shRNA-NC-HGC-27-LR and shRNA-*NONHSAT160169.1*-HGC-27-LR cells was detected by CCK-8 assay. (**i**) shRNA-NC-HGC-27-LR and shRNA-*NONHSAT160169.1*-HGC-27-LR cells were treated with lapatinib for 48 h. Cell viability was analyzed by CCK-8 assay. (**j**) Transwell assays were performed to respectively determine the migration and invasion of shRNA-NC-HGC-27-LR and shRNA-*NONHSAT160169.1*-HGC-27-LR cells. After 24 h, the cells were fixed and stained with crystal violet. Photographs were then captured and the cell count was determined (Scale bar = 100 μm). Results are shown as mean ± SEM (*p < 0.05, **p < 0.01).

To determine the role of *NONHSAT160169.1* in the development of lapatinib-induced resistance, we infected gastric cancer HGC-27 cells exhibiting low expression of *NONHSAT160169.1* with lentivirus-*NONHSAT160169.1* carrying a GFP fluorescent tag. The overexpression efficiency of *NONHSAT160169.1* in LV-*NONHSAT160169.1*-HGC-27 cells was evaluated by observing the fluorescence intensity of GFP and qPCR analysis (Fig. 3a,b). As shown in Fig. 3c,d, LV-*NONHSAT160169.1-*HGC-27 cells exhibited greater capacity for proliferation compared to LV-vector-HGC-27 cells with or without lapatinib treatment. Transwell assays suggested that the migratory and invasive abilities of the cells were enhanced following *NONHSAT160169.1* overexpression (Fig. 3e). Furthermore, we established stable *NONHSAT160169.1* knockdown cells through lentiviral infection (Fig. 3f,g). Following the knockdown of *NONHSAT160169.1*, the migration and invasive abilities of HGC-27-LR cells were reduced, thereby enhancing the sensitivity of HGC-27-LR cells to lapatinib (Fig. 3h–j). Therefore, *NONHSAT160169.1* positively regulated lapatinib resistance in HGC-27 cells.

### NONHSAT160169.1 induced stemness of HGC-27 cells by upregulating SOX2 expression

**Figure 4:**
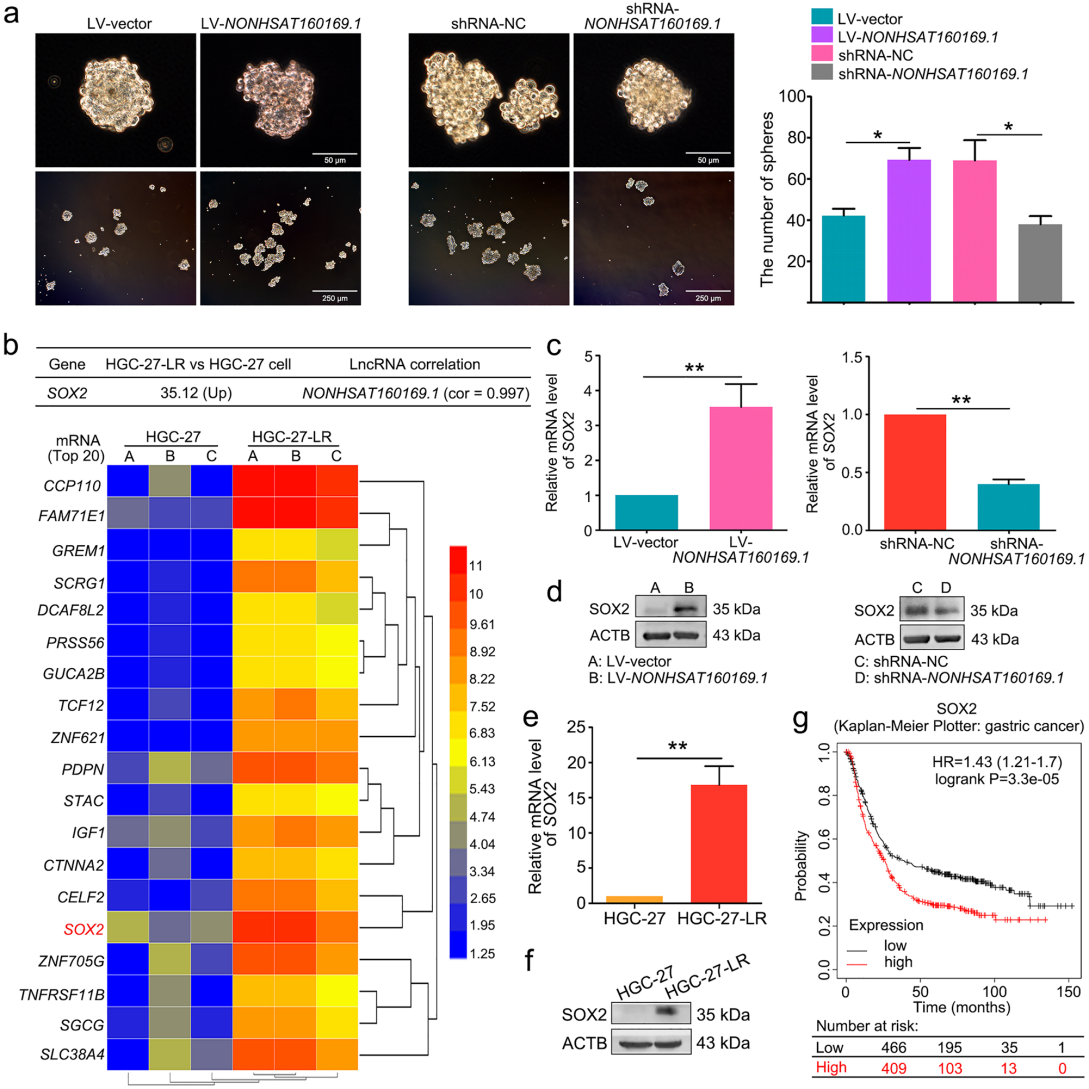
*NONHSAT160169.1* promoted the stemness of HGC-27-LR cells by upregulating the expression of SOX2. (**a**) Detection of sphere formation of LV-vector-HGC-27, LV-*NONHSAT160169.1*-HGC-27, shRNA-NC-HGC-27-LR, and shRNA-*NONHSAT160169.1*-HGC-27-LR cells after cultured in six-well ultra-low adhesion culture plates for 7 days. The number of spheres (diameter > 50 μm) was determined. (**b**) The fold-change of *SOX2* mRNA in ChIP and correlation analysis between *NONHSAT160169.1* and SOX2. A clustering heatmap of the top 20 genes upregulated at the mRNA level in HGC-27-LR cells compared to those in HGC-27 cells. The heatmap image was generated using the Heml 1.0 software: http://hemi.biocuckoo.org/down.php. (**c**,**d**) The mRNA and protein levels of SOX2 were determined by qPCR and western blot following overexpression and knockdown of *NONHSAT160169.1*. (**e**,**f**) qPCR and western blot analysis of SOX2 in HGC-27 and HGC-27-LR cells. (**g**) The relationship between SOX2 expression level and OS of in gastric cancer patients was analyzed using the Online Kaplan Meier Plotter database (https://kmplot.com/analysis/). Results are shown as mean ± SEM (*p < 0.05, **p < 0.01).

The aforementioned results indicated that an individual HGC-27-LR cell possessed a greater capacity for self-renewal and proliferation into cell clusters (Fig. 1f). Since *NONHSAT160169.1* was a key factor that promoted lapatinib resistance in HGC-27 cells, we investigated whether *NONHSAT160169.1* could affect the stem-like phenotype of cells that stably overexpress *NONHSAT160169.1* or cells in which *NONHSAT160169.1* was knocked down. The results showed that the overexpression of *NONHSAT160169.1* enhanced the stemness of HGC-27 cells, whereas the knockdown of *NONHSAT160169.1* reduced the stemness of HGC-27-LR cells (Fig. 4a).

To understand the mechanisms by which *NONHSAT160169.1* promotes HGC-27-LR cell stemness, we performed transcriptome microarray sequencing of all coding genes in both HGC-27 and HGC-27-LR cells. Among the top 20 notably upregulated coding genes in HGC-27-LR cells, SOX2 attracted our attention, which was positively correlated with *NONHSAT160169.1* (Fig. 4b). Studies have shown that SOX2 promotes tumor cell stemness and plays a crucial role in triggering resistance to cancer therapies^20–23^. We hypothesized that *NONHSAT160169.1* increased lapatinib resistance in HGC-27 cells by inducing SOX2 expression. qPCR and western blot analysis revealed that mRNA and protein levels of SOX2 were increased in HGC-27 cells following *NONHSAT160169.1* overexpression. Similarly, the expression of SOX2 was decreased in HGC-27-LR cells in which *NONHSAT160169.1* was knocked down (Fig. 4c,d). The expression of SOX2 was then compared between HGC-27 and HGC-27-LR cells. The results showed that the expression of SOX2 was increased in HGC-27-LR cells (Fig. 4e,f). Additionally, using the Online Kaplan Meier Plotter database (https://kmplot.com/analysis/), we found that high expression of SOX2 was positively correlated with short OS in gastric cancer patients. Investigating the effect of SOX2 on lapatinib-resistant HGC-27 cells may hold some clinical significance (Fig. 4g). Hence, these results indicated that *NONHSAT160169.1* induced stemness in HGC-27 cells by upregulating SOX2 expression.

### NONHSAT160169.1 increased SOX2 expression by decoying hsa-let-7c-3p as a sponge RNA

**Figure 5:**
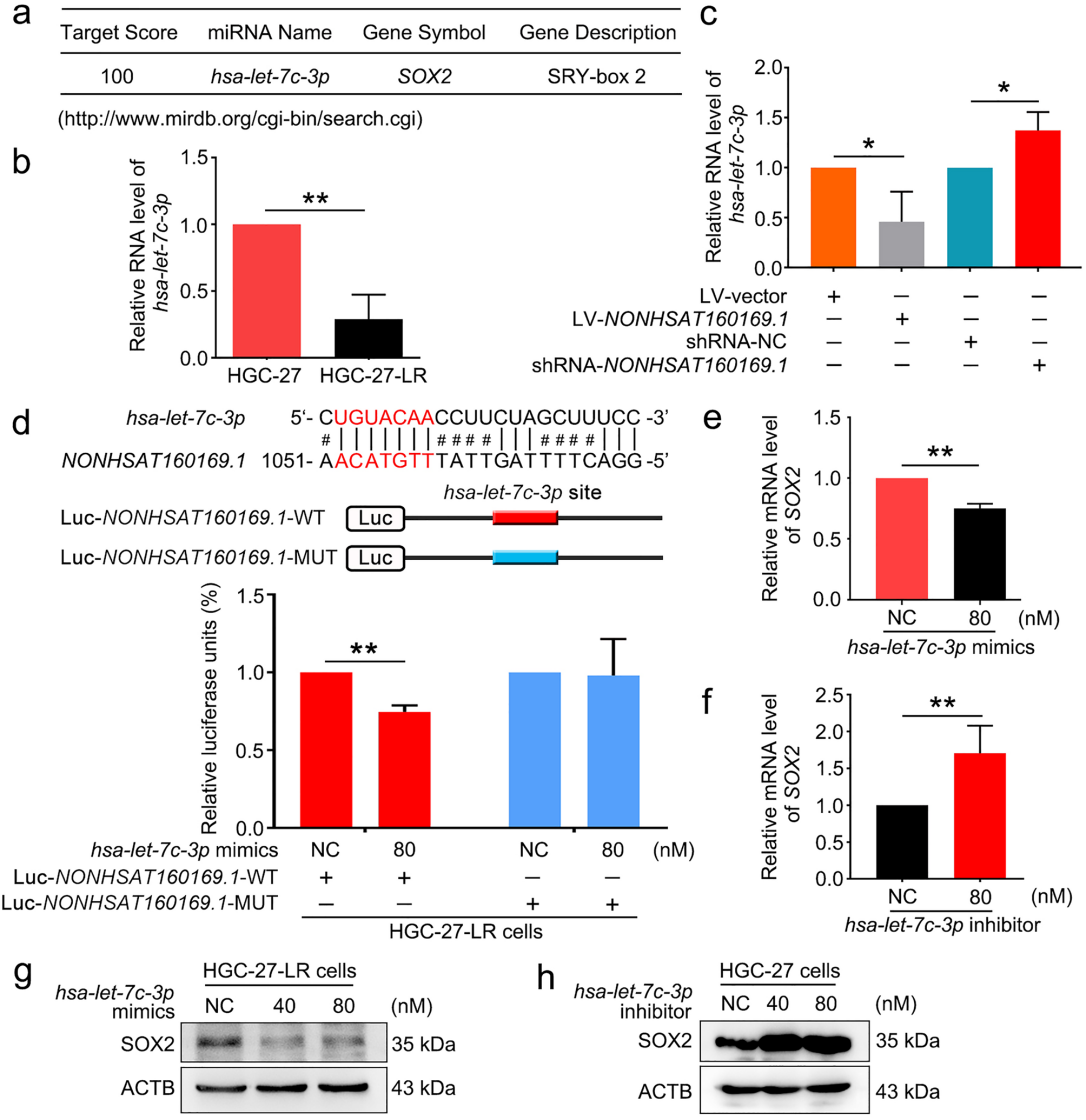
*NONHSAT160169.1* increased SOX2 expression by directly binding to *hsa-let-7c-3p*. (**a**) The target gene for *hsa-let-7c-3p* was predicted using a bioinformatics prediction website (http://www.mirdb.org/cgi-bin/search.cgi). (**b**) qPCR analysis of *hsa-let-7c-3p* in HGC-27 and HGC-27-LR cells. (**c**) qPCR analysis of *hsa-let-7c-3p* following overexpression and knockdown of *NONHSAT160169.1*. (**d**) Luc-*NONHSAT160169.1-*WT or Luc-*NONHSAT160169.1*-MUT plasmid was co-transfected with NC or *hsa-let-7c-3p* mimics into HGC-27-LR cells for 24 h. Luciferase activity was determined. (**e**) Following transfection with NC and *hsa-let-7c-3p* mimics for 72 h, we performed qPCR analysis of the mRNA level of *SOX2* in HGC-27-LR cells. (**f**) HGC-27 cells were transfected with *hsa-let-7c-3p* inhibitor for 72 h. The mRNA level of *SOX2* was analyzed by qPCR. (**g**) Following transfection with NC and *hsa-let-7c-3p* mimics for 72 h, we performed western blot analysis of the protein level of SOX2 in HGC-27-LR cells. (**h**) HGC-27 cells were transfected with *hsa-let-7c-3p* inhibitor for 96 h. Western blot analysis of SOX2. Results are shown as mean ± SEM (*p < 0.05, **p < 0.01).

Studies have shown that lncRNAs can sponge miRNAs and subsequently affect the stability of target mRNA as competing endogenous RNAs (ceRNAs)^24^. Initially, we predicted the miRNAs that could potentially target *SOX2* mRNA using the miRDB-MicroRNA Target Prediction and Functional Study Database (http://www.mirdb.org/cgi-bin/search.cgi). The top-ranked predicted miRNA was *hsa-let-7c-3p*, with a prediction score of 100 (Fig. 5a). Thus, we compared *hsa-let-7c-3p* levels in both HGC-27 and HGC-27-LR cells. qPCR showed that the level of *hsa-let-7c-3p* was significantly reduced in HGC-27-LR cells (Fig. 5b). Moreover, the RNA level of *hsa-let-7c-3p* was decreased in LV-*NONHSAT160169.1*-HGC-27 cells; however, it increased following *NONHSAT160169.1* knockdown (Fig. 5c).

To validate the direct interaction of *hsa-let-7c-3p* and *NONHSAT160169.1*, we predicted the potential binding sites between *hsa-let-7c-3p* and *NONHSAT160169.1*. The results showed that the putative sites were located at positions 1052 to 1059 bp. Luciferase assays showed that co-transfection of Luc-*NONHSAT160169.1*-WT plasmid with *hsa-let-7c-3p* mimics into HGC-27-LR cells significantly repressed luciferase activity compared to transfection of Luc-*NONHSAT160169.1*-MUT or negative control (NC) (Fig. 5d). Thus, these results showed that *NONHSAT160169.1* interacted directly with *hsa-let-7c-3p*.

Next, we determined the effect of *hsa-let-7c-3p* on the expression of SOX2. The mRNA and protein levels of SOX2 decreased following transfection of *hsa-let-7c-3p* mimics into HGC-27-LR cells exhibiting high SOX2 expression (Fig. 5e,g); however, mRNA and protein levels of SOX2 increased following transfection of *hsa-let-7c-3p* inhibitor into HGC-27 cells (Fig. 5f,h). Therefore, these results validated that *hsa-let-7c-3p* was negatively modulated and *NONHSAT160169.1* promoted the expression of SOX2.

### SOX2 overexpression promoted migration, invasion, self-renewal, and lapatinib resistance in HGC-27 cells

To investigate whether SOX2 overexpression altered the sensitivity of HGC-27 cells to lapatinib, we stably overexpressed SOX2. Overexpression of SOX2 was observed using a GFP fluorescent tag and validated through western blot analysis (Fig. S1a). Transwell assays suggested that the migratory and invasive abilities of HGC-27 cells were enhanced after SOX2 overexpression, with or without lapatinib treatment (Fig. S1b,c). Additionally, the sphere formation assay indicated that LV-SOX2-HGC-27 cells possessed a greater capacity for self-renewal compared to LV-vector-HGC-27 cells (Fig. S1d). Moreover, following treatment with lapatinib at various concentrations for 72 h, LV-SOX2-HGC-27 cells were more viable compared to LV-vector-HGC-27 cells (Fig. S1e). Therefore, SOX2 upregulation induced by *NONHSAT160169.1* adsorbing *hsa-let-7c-3p* contributed to the activation of lapatinib resistance in HGC-27-LR cells.

### Lapatinib-induced p-STAT3 upregulation promoted the transcription of NONHSAT160169.1 and lapatinib tolerance in HGC-27 cells

**Figure 6:**
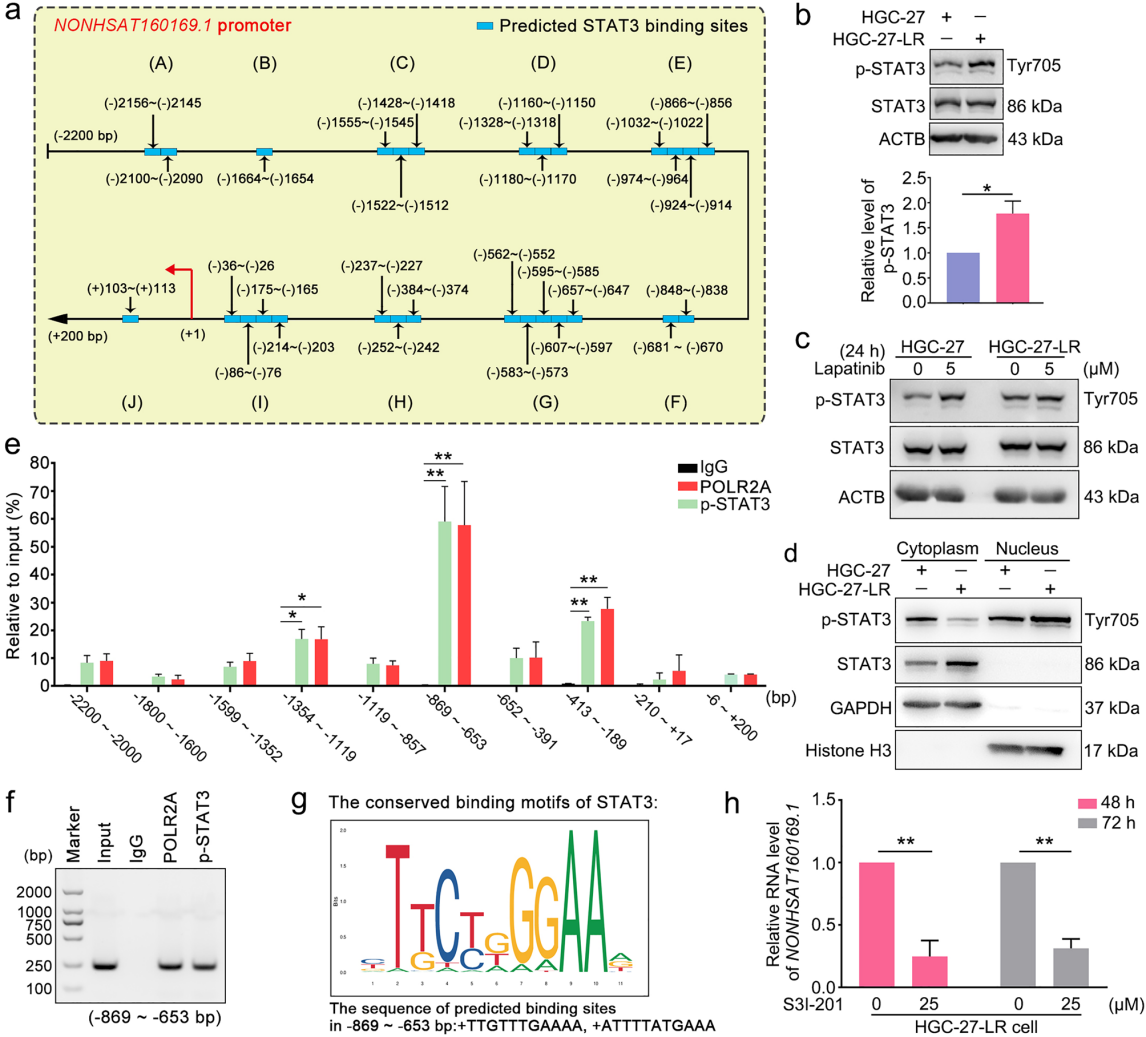
Lapatinib-induced increase in the level of p-STAT3 promoted *NONHSAT160169.1* transcription. (**a**) Using the JASPAR database, we predicted multiple binding sites of STAT3 in *NONHSAT160169.1* promoter. (**b**) Western blot analysis of p-STAT3 and STAT3 in HGC-27 and HGC-27-LR cells. The gray values of the bands were analyzed. (**c**) HGC-27 and HGC-27-LR cells were respectively treated with 5 μM lapatinib for 24 h. Western blot analysis of the protein levels of p-STAT3 and STAT3. The gray values of the bands were analyzed. (**d**) Western blot analysis of p-STAT3 in the subcellular fractions of HGC-27 and HGC-27-LR cells (GAPDH: cytoplasmic protein marker; histone H3: nucleus protein marker). (**e**) ChIP analysis of the direct binding of p-STAT3 to *NONHSAT160169.1* promoter detected by using qPCR. (**f**) Agarose gel electrophoresis of the qPCR product of the most significant DNA fragment obtained from (**e**) (− 869 ~ − 653 bp). (**g**) The predicted binding motifs of STAT3 corresponding to the *NONHSAT160169.1* promoter are shown. The sequence of predicted binding sites in − 869 ~ − 653 bp were + TTGTTTGAAAA and + ATTTTATGAAA. (**h**) In HGC-27-LR cells, the RNA level of *NONHSAT160169.1* was detected using qPCR following treatment with S3I-201 for 48 h and 72 h. Results are shown as mean ± SEM (*p < 0.05, **p < 0.01).

We further explored the mechanism underlying *NONHSAT160169.1* upregulation in HGC-27-LR cells. Through bioinformatics analysis (http://jaspar.binf.ku.dk/), we predicted that the STAT3 could bind to the promoter of *NONHSAT160169.1*. Potential sites in the *NONHSAT160169.1* promoter where STAT3 can bind are shown in Fig. 6a. Previous studies have validated that STAT3 is a key downstream signal molecule of EGFR/HER2 and is involved in the regulation of chemoresistance^25,26^. p-STAT3 translocates into the nucleus after phosphorylation and then functions as a transcription factor to mediate the expression of its target genes^27^. Thus, we compared STAT3 and p-STAT3 levels in HGC-27 and HGC-27-LR cells. Western blot analysis revealed that p-STAT3 was markedly increased in HGC-27-LR cells (Fig. 6b). This phenomenon was induced by lapatinib treatment, as the results suggested that lapatinib significantly upregulated the level of p-STAT3 in HGC-27 cells but not HGC-27-LR cells (Fig. 6c). Moreover, analysis of the proteins in the nuclear and cytoplasmic fractions suggested that, compared to that in HGC-27 cells, the nuclear distribution of p-STAT3 in the HGC-27-LR cells was increased (Fig. 6d). Therefore, the increase in *NONHSAT160169.1* expression induced by lapatinib was dependent on nuclear translocation of phospho-STAT3 (Tyr705).

We designed 10 pairs of various primers according to the predicted binding sites. ChIP and qPCR analysis validated that could bind most strongly to a − 869 ~ − 653 bp fragment of *NONHSAT160169.1* promoter (Fig. 6e). Moreover, agarose gel electrophoresis of − 869 ~ − 653 bp qPCR products showed discernable and distinct bright bands (Fig. 6f). Predicted STAT3 binding site motifs were highly consistent with the predicted sites on the *NONHSAT160169.1* promoter (Fig. 6g). Furthermore, to comprehensively understand whether STAT3 was the upstream signaling molecule of *NONHSAT160169.1*-induced SOX2 upregulation, we treated HGC-27-LR cells with S3I-201, which is an inhibitor of STAT3 DNA binding activity. Subsequently, the total RNA from HGC-27-LR cells was extracted, and expression levels of *NONHSAT160169.1* and SOX2 were determined. The results showed that S3I-201 treatment dramatically decreased the level of *NONHSAT160169.1* (Fig. 6h), and the mRNA and protein levels of downstream SOX2 were also significantly downregulated (Fig. S2a,b).

Furthermore, we conducted a comprehensive investigation on the intricate interplay between STAT3 and SOX2 expression, a downstream molecule of *NONHSAT160169.1*, and the role of STAT3 in regulating the sensitivity of HGC-27 cells to lapatinib by constructing stable overexpression (LV-vector-HGC-27 and LV-STAT3-HGC-27) and knock-down STAT3 (sh-NC-HGC-27-LR and sh-STAT3-HGC-27-LR) cell lines. The overexpression and knockdown efficiency of STAT3 were detected by western blot. The results suggested that the expression of SOX2 increased in LV-STAT3-HGC-27 cells, while in the sh-STAT3-HGC-27-LR cells, the expression of SOX2 reduced (Fig. S3). We next performed CCK-8 analysis to clarify the effect of STAT3 on lapatinib resistance in HGC-27 cells. The results showed that lapatinib sensitivity of HGC-27-LR cells was increased after STAT3 knockdown (Fig. S4a,b). In addition, after overexpression of STAT3, the viability of HGC-27 cell was higher than that of empty vector transfection group under the treatment of different concentrations of lapatinib for 72 h (Fig. S4c,d). The above results suggested that STAT3 aggravated lapatinib tolerance in HGC-27 cells.

Therefore, p-STAT3 was the upstream inducer of *NONHSAT160169.1*. Long-term lapatinib treatment increased the level of p-STAT3 in HGC-27 cells, accompanied by its nuclear translocation, allowing it to bind to the *NONHSAT160169.1* promoter and induce its expression. Moreover, the expression of SOX2 downstream of *NONHSAT160169.1* was increased.

### NONHSAT160169.1 promoted the progression of HGC-27 xenografts in NCG mouse model

**Figure 7:**
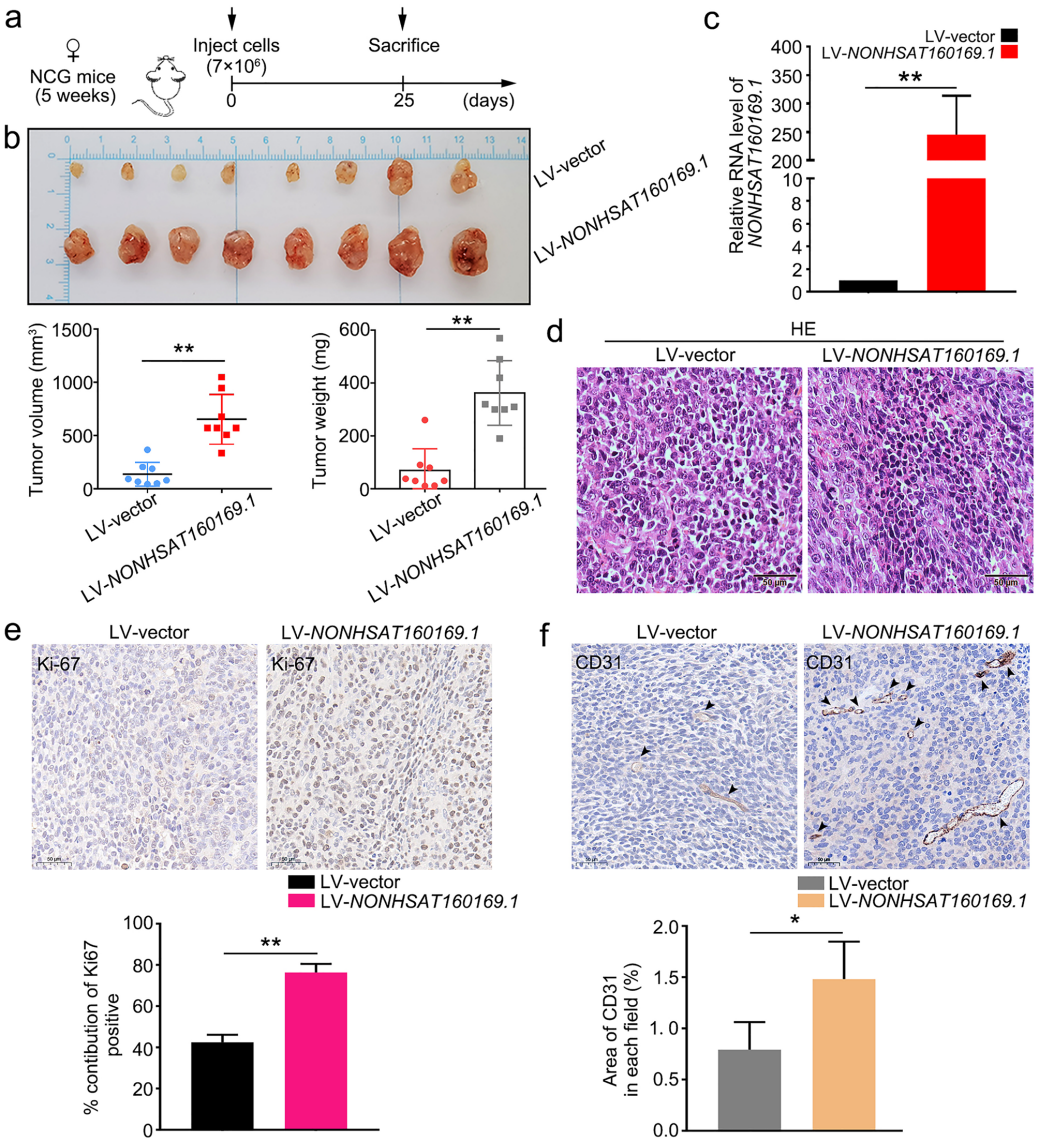
Overexpression of *NONHSAT160169.1* promoted the growth of HGC-27 xenografts in vivo. (**a**) Design of the experiments involving LV-vector and LV-*NONHSAT160169.1*-HGC-27 xenografts. NCG mice were randomly divided into two groups. NCG mice were subcutaneously injected with LV-vector-HGC-27 and LV-*NONHSAT160169.1*-HGC-27 cells (7 × 10^6^). (**b**) Images of the xenograft tumors obtained from NCG mice at the endpoint (n = 8). The volume and weight of the xenograft tumors from each group at the endpoint. (**c**) qPCR analysis of *NONHSAT160169.1* level in xenograft tumors. (**d**–**f**) HE, Ki-67, and CD31 staining for IHC of the xenograft tumors. Results are shown as mean ± SEM (**p < 0.01).

We next examined the effect of *NONHSAT160169.1* on the progression of HGC-27 xenograft tumors in vivo. NCG mice were subcutaneously injected with LV-vector-HGC-27, LV-*NONHSAT160169.1*-HGC-27, shRNA-NC-HGC-27-LR, or shRNA-*NONHSAT160169*.1-HGC-27-LR cells (7 × 10^6^). We monitored the volumes of the subcutaneous xenografts every 2 days. At the endpoint, the xenograft tumors were obtained from the NCG mice and photographed (Fig. 7a,b).

**Figure 8:**
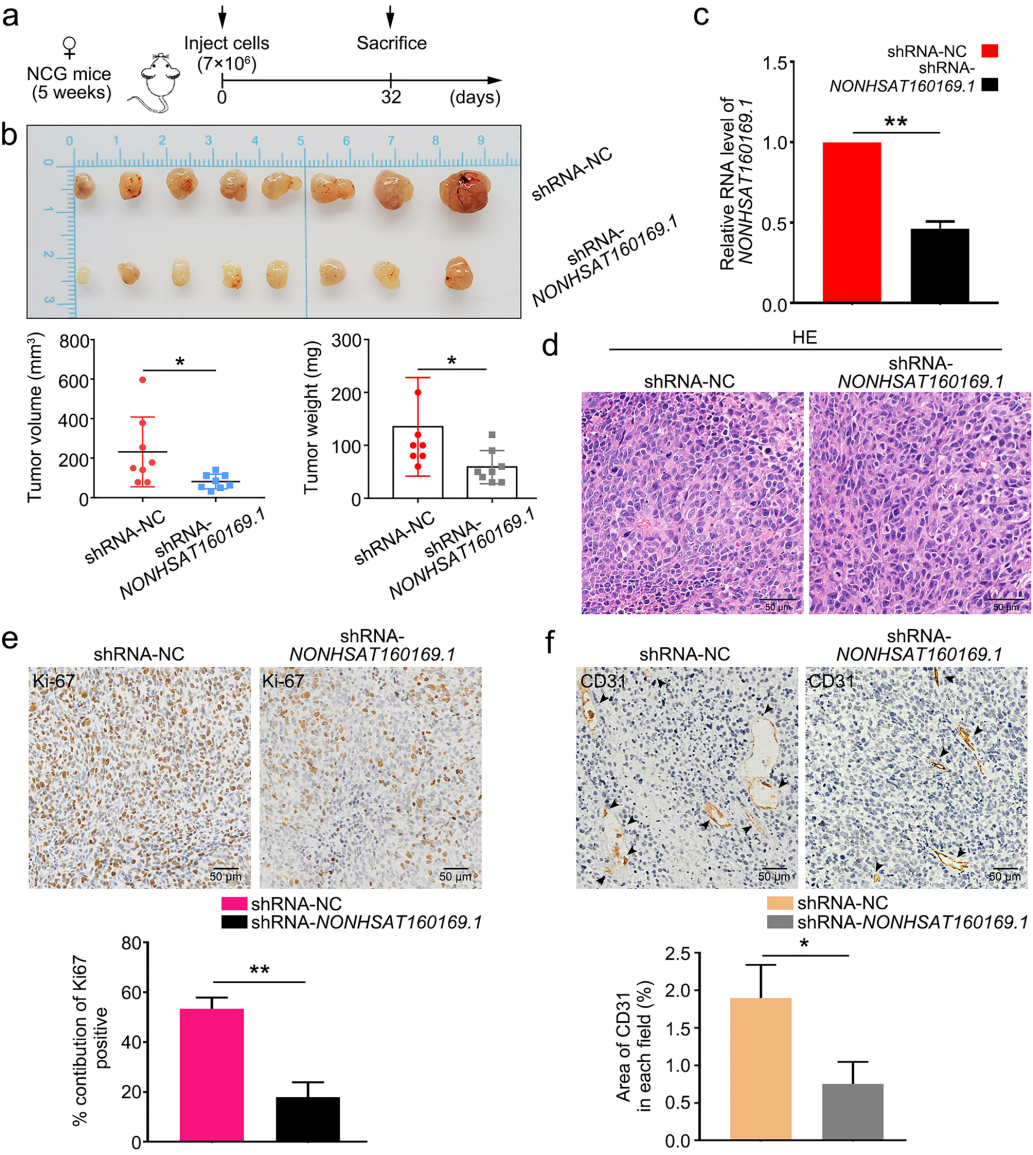
Knockdown of *NONHSAT160169.1* inhibited the growth of HGC-27-LR xenografts in vivo. (**a**) Design of the experiments involving shRNA-NC and shRNA-*NONHSAT160169.1*-HGC-27-LR xenografts. NCG mice were randomly divided into two groups. NCG mice were subcutaneously injected with shRNA-NC and shRNA-*NONHSAT160169.1*-HGC-27-LR cells (7 × 10^6^). (**b**) Images of the xenograft tumors obtained from NCG mice at the endpoint (n = 8). The volume and weight of the xenograft tumors from each group at the endpoint. (**c**) qPCR analysis of *NONHSAT160169.1* level in the xenograft tumors. (**d**–**f**) HE, Ki-67, and CD31 staining for IHC of the xenograft tumors. Results are shown as mean ± SEM (*p < 0.05, **p < 0.01).

In comparation with those in the LV-vector group, the volume and weight of the LV-*NONHSAT160169.1*-HGC-27 xenografts markedly increased (Fig. 7b,c). Furthermore, the knockdown of *NONHSAT160169.1* significantly inhibited the growth of HGC-27-LR xenograft tumors (Fig. 8b,c), consistent with the results of in vitro experiments. After embedding xenograft tumor tissues in paraffin and preparing tissue sections, hematoxylin–eosin (HE), Ki-67, and CD31 (platelet and endothelial cell adhesion molecule 1) staining were performed for IHC. The results showed that overexpression of *NONHSAT160169.1* was accompanied by high levels of Ki-67 and CD31 in xenograft tumor tissues (Fig. 7e,f). Moreover, these levels decreased after *NONHSAT160169.1* knockdown (Fig. 8e,f).

## Discussion

Amplification of the HER2 oncogene occurs in approximately 17%–20% of patients with gastric cancer^28^. Several clinical trials of lapatinib for HER2 target therapy have been conducted for the treatment of patients with advanced gastric cancer. However, these clinical trials have yielded unsatisfactory results^29,30^. One of the primary factors limiting the efficacy and clinical application of tumor-targeted therapies is drug resistance. The dysregulation of lncRNAs plays a crucial role in resistance to targeted anti-tumor therapy^31–33^. To date, few lncRNAs have been implicated in the regulation of lapatinib-induced tumor resistance, particularly in gastric cancer. And the mechanism of lapatinib resistance in HER2-positive gastric cancer remains unclear. Thus, we aimed to identify novel lncRNAs and the potential signaling pathways involved in adaptive lapatinib resistance in HER2-positive gastric cancer.

In this study, HGC-27 cells resistant to lapatinib (HGC-27-LR cells) were established through a classical approach involving long-term drug gradient pressure in vitro. HGC-27-LR cells exhibited enhanced migration, invasion, and stemness phenotypes (Fig. 1). Gene chip analysis and experimental validation revealed that a novel lncRNA, namely *NONHSAT160169.1*, was most significantly upregulated in HGC-27-LR cells and that it is distributed in both the cytoplasm and nucleus (Fig. 2). At present, there is no evidence regarding the function of *NONHSAT160169.1*. Our study showed that *NONHSAT160169.1* positively regulates lapatinib resistance in HGC-27-LR cells by promoting cell migration, invasion, and stemness (Fig. 3). To investigate the mechanism underlying *NONHSAT160169.1-*induced lapatinib resistance in HGC-27-LR cells, we performed transcriptome sequencing of coding genes. As one of the crucial genes regulating cell stemness, the significant upregulation of *SOX2* in HGC-27-LR cells attracted our attention. Moreover, we predicted that *SOX2* expression was positively correlated with *NONHSAT160169.1* (correlation coefficient = 0.997), and additional experiments revealed that *NONHSAT160169.1* enhanced *SOX2* expression (Fig. 4).

A complex regulatory network consisting of lncRNAs, miRNAs, and mRNA targets represents a form of classical gene expression regulation. The crosstalk between them serves an important role in tumor therapy resistance^34,35^. Hence, through bioinformatics prediction, we searched for miRNAs that could bind directly to *SOX2* 3′UTR and *NONHSAT160169.1*. The results suggested that the *hsa-let-7c-3p* level was reduced in HGC-27-LR cells and that *NONHSAT160169.1* may decoy *hsa-let-7c-3p*. Additional experiments showed that *hsa-let-7c-3p* targets and negatively regulates the expression of SOX2 (Fig. 5). This study is the first to elucidate that SOX2 is a target gene for *hsa-let-7c-3p*.

Subsequently, we investigated whether the upregulation of SOX2 expression induced by *NONHSAT160169.1* sponging *hsa-let-7c-3p* could enhance lapatinib resistance in HGC-27 cells. The results indicated that the overexpression of SOX2 in HGC-27 cells positively regulated lapatinib resistance by promoting cell stemness, migration, and invasion (Fig. S1). The aberrant expression of SOX2 has been widely reported to positively correlate with cell proliferation, stemness, metastasis, tumorigenesis, and chemotherapy resistance, in various cancers^36,37^. However, the relationship between SOX2 expression and lapatinib resistance in HER2-positive gastric cancer cells remains unclear. On the basis of the aforementioned results, we hypothesized that *NONHSAT160169.1* could increase the expression of SOX2 in HGC-27-LR cells and contribute to lapatinib resistance by decoying *hsa-let-7c-3p*.

Next, we investigated the mechanism underlying the upregulation of *NONHSAT160169.1* in lapatinib-resistant gastric cancer cells. Prediction results from bioinformatics analysis (http://jaspar.binf.ku.dk/) suggested that the *NONHSAT160169.1* promoter may contain multiple potential binding sites for STAT3 (Fig. 6a). Generally, phosphorylated STAT3 can function as a transcription factor to modulate the expression of its target genes. We hypothesized that p-STAT3 in HGC-27-LR cells may translocate into the nucleus and bind to the *NONHSAT160169.1* promoter to initiate transcription. Western blot showed that the level of p-STAT3 was higher in HGC-27-LR cells than in HGC-27 cells due to prolonged lapatinib stimulation (Fig. 6b,c). Moreover, we observed that the level of p-STAT3 exhibiting nuclear transport was significantly higher in HGC-27-LR cells than in maternal cells (Fig. 6d). CHIP experiments demonstrated that p-STAT3 combined directly to the promoter of *NONHSAT160169.1* (Fig. 6e–g). Moreover, the levels of *NONHSAT160169.1* and SOX2 were notably decreased after inhibiting the DNA-binding activity of STAT3. In particular, we focused on the effect of STAT3 regulation on SOX2 expression and the sensitivity of HGC-27 cells to lapatinib. The results demonstrated that the overexpression of STAT3 in HGC-27 cells could significantly increase the level of SOX2 and enhance the resistance to lapatinib (Figs. S3–S4).

Therefore, lapatinib-induced nuclear translocation of the transcription factor p-STAT3 promoted the transcription of *NONHSAT160169.1* and activated the *hsa-let-7c-3p/SOX2* signaling axis. Notable increases in tumor volume and weight in vivo were observed following *NONHSAT160169.1* overexpression, whereas following *NONHSAT160169.1* knockdown, the growth of HGC-27-LR xenograft tumors was significantly inhibited (Figs. 7, 8).

**Figure 9:**
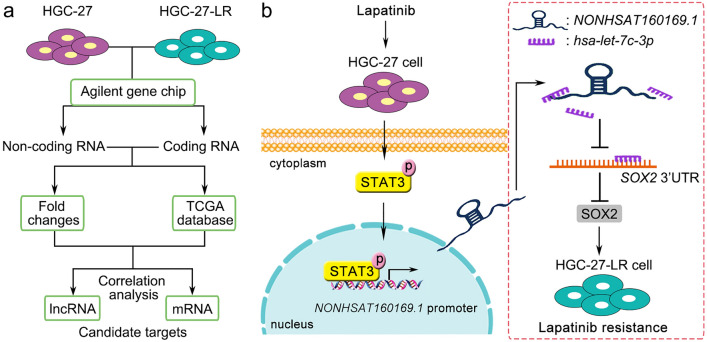
Schematic of the mechanism by which *NONHSAT160169.1* promoted resistance in gastric cancer cells. (**a**, **b**) On the basis of the noncoding and coding transcriptome sequencing analyses, we identified key factors involved in the regulation of lapatinib resistance in HGC-27 cells. Long-term treatment with lapatinib promoted p-STAT3 nuclear translocation, thereby upregulating the expression of *NONHSAT160169.1*, which then enhanced the migration, invasion, and stemness phenotypes of HGC-27 cells. *NONHSAT160169.1* decoyed *hsa-let-7c-3p* as a ceRNA and contributed to reversing the inhibitory effect of *hsa-let-7c-3p* on SOX2 expression. Therefore, *NONHSAT160169.1*/*hsa-let-7c-3p*/SOX2 was shown to be a novel signaling pathway positively regulating lapatinib resistance in HGC-27 cells.

In conclusion, we elucidated a novel mechanism underlying lapatinib resistance in gastric cancer from the perspectives of lncRNA, miRNA, and mRNA using genome-wide gene chip technology for high-throughput screening. Long-term lapatinib stimulation upregulates the expression of *NONHSAT160169.1 *via p-STAT3 (Tyr705) in HGC-27-LR cells. As a ceRNA, *NONHSAT160169.1* decoys *hsa-let-7c-3p* and increases the level of SOX2, thereby aggravating lapatinib resistance in HGC-27 gastric cancer cells (Fig. 9). Above all, our study elucidates a novel survival signaling pathway during the progression of adaptive lapatinib resistance in HGC-27 cells, and targeting *NONHSAT160169.1* is expected to be a potential therapeutic strategy for overcoming lapatinib resistance in HER2-positive gastric cancer.

### Supplementary Information

Supplementary Information 1.

Supplementary Information 2.

Supplementary Information 3.

Supplementary Information 4.

Supplementary Information 5.

## Data Availability

All data and materials are availability within the article, or available from the corresponding authors upon request.

## Abbreviations

HER2: ERBB2 receptor tyrosine kinase 2
HGC-27-LR: Lapatinib-resistant cells derived from HGC-27
lncRNA: Long noncoding RNA
STAT3: Signal transducer and activator of transcription 3
SOX2: SRY-box transcription factor 2
OS: Overall survival
IC_50_: Half-maximal inhibitory concentration
RI: Resistance index
CD133: Prominin 1
ALDH1A1: Aldehyde dehydrogenase 1 family member A1
LV: Lentivirus
miRNA: MicroRNA
ceRNAs: Competing endogenous RNAs
MUT: Mutation
WT: Wild type
NC: Negative control
qPCR: Real-time Quantitative PCR Detecting System
ChIP: Chromatin Immunoprecipitation
HE: Hematoxylin–eosin
CD31: Platelet and endothelial cell adhesion molecule 1
