# Supplementary figures and images for: Long noncoding RNA NONHSAT160169.1 promotes resistance via hsa-let-7c-3p/SOX2 axis in gastric cancer
Source: Sci Rep. 2023 Nov 27;13:20858. doi: 10.1038/s41598-023-47961-5 (PMC10682003; doi:10.1038/s41598-023-47961-5)

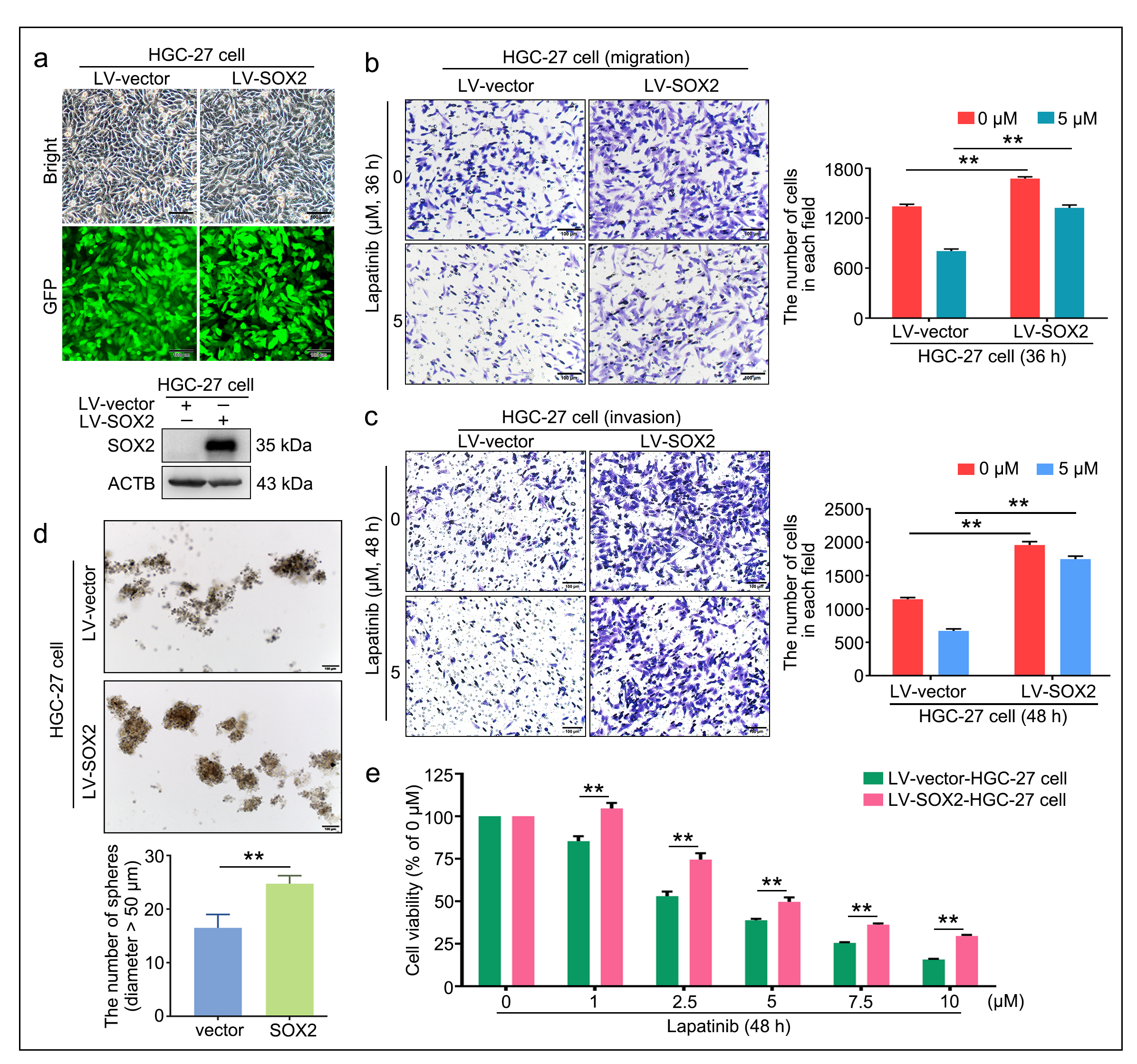

Supplement: Supplementary file 1 — Supplementary Information 1. [file 41598_2023_47961_MOESM1_ESM.jpg]

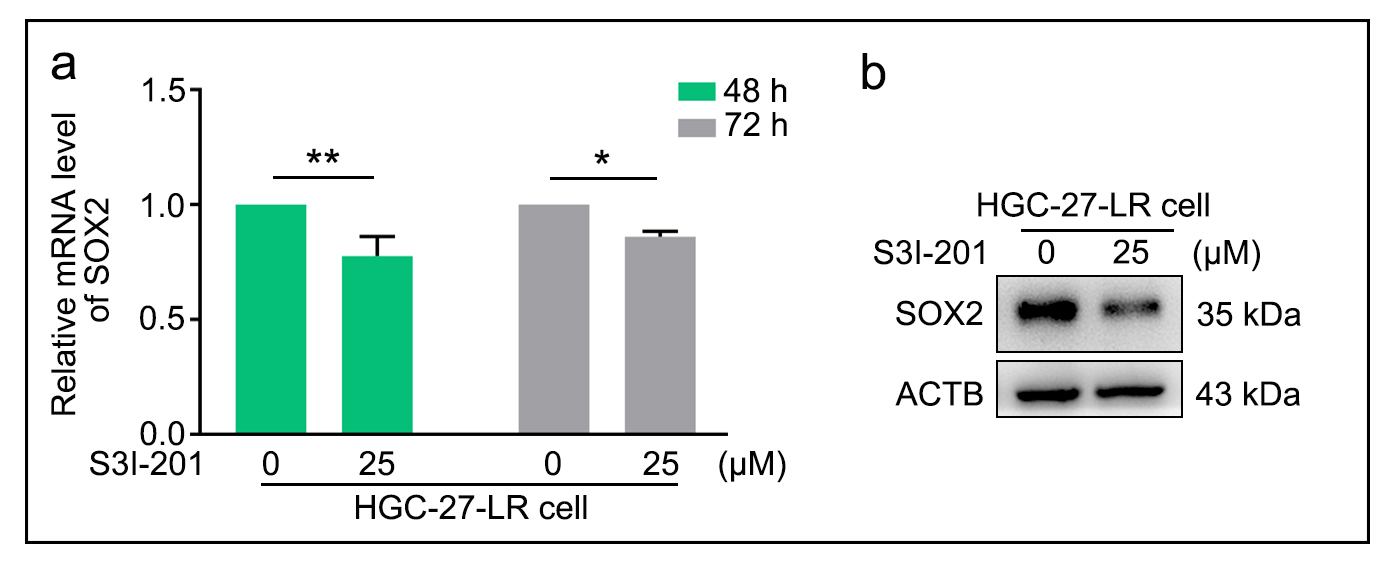

Supplement: Supplementary file 2 — Supplementary Information 2. [file 41598_2023_47961_MOESM2_ESM.jpg]

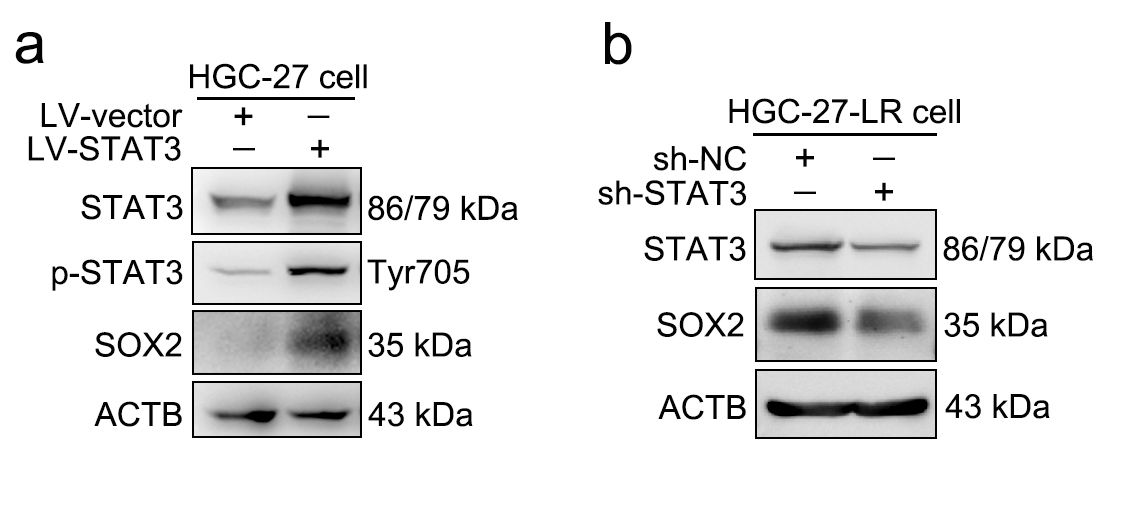

Supplement: Supplementary file 3 — Supplementary Information 3. [file 41598_2023_47961_MOESM3_ESM.jpg]

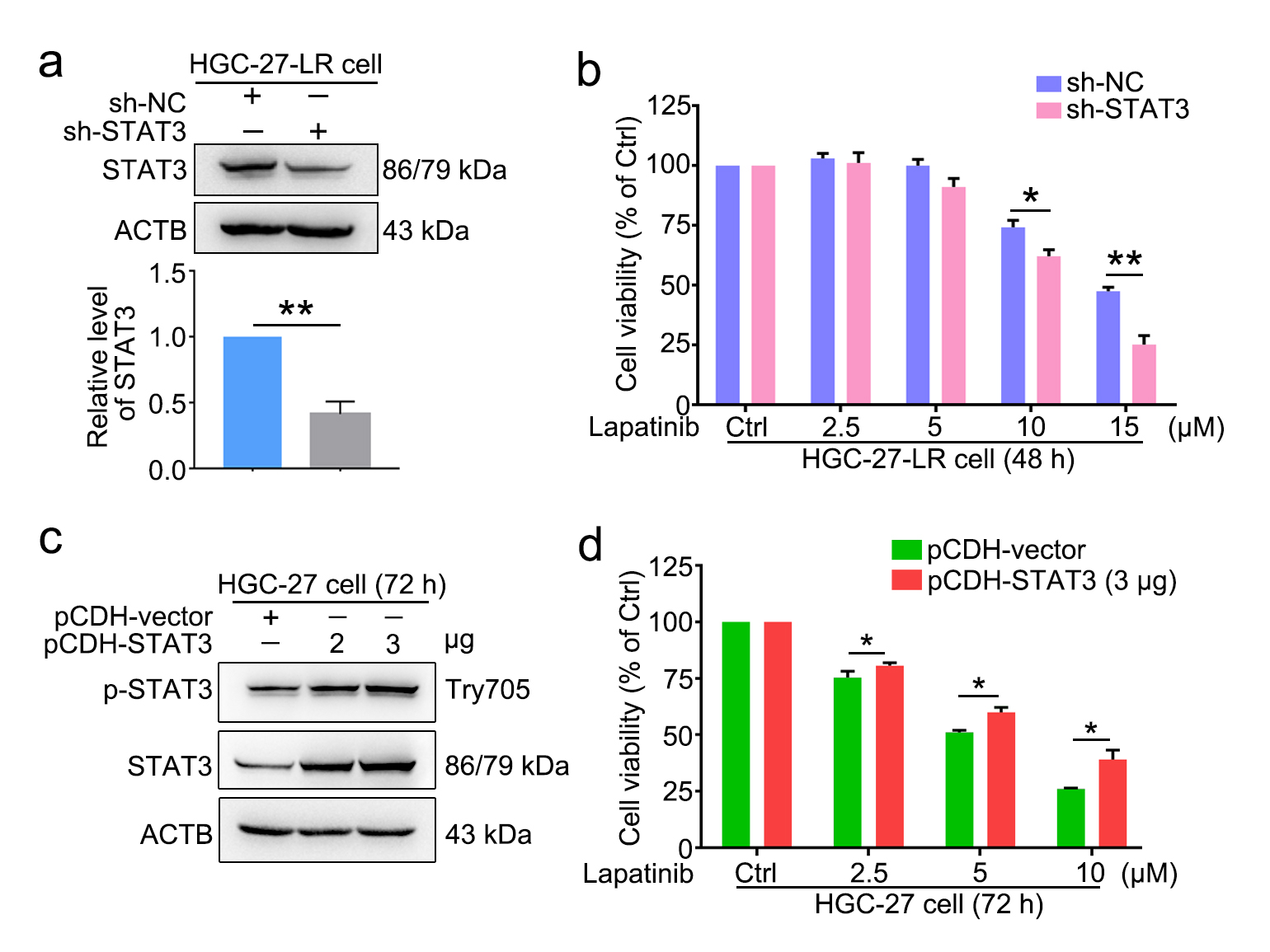

Supplement: Supplementary file 4 — Supplementary Information 4. [file 41598_2023_47961_MOESM4_ESM.jpg]
