# Supplementary material for: Long noncoding RNA NONHSAT160169.1 promotes resistance via hsa-let-7c-3p/SOX2 axis in gastric cancer
Source: Sci Rep. 2023 Nov 27;13:20858. doi: 10.1038/s41598-023-47961-5 (PMC10682003; doi:10.1038/s41598-023-47961-5)
Supplement: Supplementary file 5 — Supplementary Information 5. [file 41598_2023_47961_MOESM5_ESM.pdf]

Supplementary information for:

Long noncoding RNA *NONHSAT160169.1* promotes resistance via *hsa-let-7c-3p*/SOX2 axis in gastric cancer

Xuan Zhao[1,2,3†], Zijian Xu[1,2,3†], Bi Meng[1,2,3], Tong Ren[1,2,3], Sijin Li[1,2,3], Xu Wang[1,2,3], Rui Hou[4], Wen Ma[1,2,3], Dan Liu[1,2,3]*, Junnian Zheng[2,3]*, Ming Shi[1,2,3]*

1. Cancer Institute, Xuzhou Medical University, 209 Tongshan Road, Xuzhou, Jiangsu, 221004, China
2. Center of Clinical Oncology, The Affiliated Hospital of Xuzhou Medical University, 99 Huaihai Road, Xuzhou, Jiangsu, 221002, China
3. Jiangsu Center for the Collaboration and Innovation of Cancer Biotherapy, Xuzhou Medical University, 209 Tongshan Road, Xuzhou, Jiangsu, 221004, China
4. College of Pharmacy, Xuzhou Medical University, 209 Tongshan Road, Xuzhou, Jiangsu, 221004, China

† These authors contributed equally to this work and share first authorship.
Correspondence to Ming Shi, email: sm200@sohu.com.

Supplementary Materials and Methods：

Supplementary Tables:

Table S1. List of primer sequences.

Gene name	Forward primer	Reverse primer
ACTB	GAAGTGTGACGTGGACATCC	CCGATCCACACGGAGTACTT
NONHSAT160169.1	GCATGGGCAGTCTCTTTCGT	CACTGAGGCTGATTTGAGGGA
SOX2	GCCGAGTGGAAACTTTTGTCG	GGCAGCGTGTACTTATCCTTCT
hsa-let-7c-3p	TCCGCCTGTACAACCTTCTAG	AGAGCAGGGTCCGAGGT

Table S2. List of *hsa-let-7c-3p* mimics and inhibitor sequences.

Name	Sense
NC-mimics-sense	UUCUCCGAACGUGUCACGUTT
NC-mimics-antisense	ACGUGACACGUUCGGAGAATT
mimics-sense	CUGUACAACCUUCUAGCUUUCC
mimics-antisense	AAAGCUAGAAGGUUGUACAGUU
NC-inhibitor	CAGUACUUUUGUGUAGUACAA
inhibitor	GGAAAGCUAGAAGGUUGUACAG

Table S3. The sequence of *NONHSAT160169.1*.

>*NONHSAT160169.1*
Length 1384bp (database: NONCODE)
GGAGTATTGCTTGAATCCAGGAGAGGGAGGTTGCTGTGAGCCTAGA<br>TCGCACCACTGCAGTCCAGCCTGGGTGACAGAGTAAGACCTTTTCT<br>CAAAAAAAGAAAAATATGTAAATAAATAAGATGTAAGTTGGACATC<br>AATAGGGCTGTGGAAGAAAAGAAACAGAGGAAGCATGGGAAAGC<br>AGAAGCAGGCAGCACTCCTGTTTCTATGGGTATTTTCTCCTTCTTCC<br>TTGAAATGTAACTGAAACATTCATGTTGACTTGCCAGTGTTCCATAC<br>CTATCTCCCTCCCCAAGTAATAAGGGACTGGCTTCATTCAGACTCAC<br>AGTGCCACACTGTTCTGGGACAGTTATACTCATTGTCATCACTTTCA<br>TGGATATAAACAACCCAGACTCCTGCAGAGAAAATGAGAAGTCTGG<br>TTAAGTAACTTGATATTTTGCTGATGTGGTATCACATGCATGGAGTTT<br>CTGAAGCATGTTGACTGCACATTTTCATTCTTATCCAGATACTTAGCA<br>TCATGGAAACATAGACCAAAGGAGTTTTCTTACTGTTTATGCCAAGT<br>GGAAGACCAAGGCCTATTTTCACTCTATCCCAGAAGAAAACTACAA<br>CTTCCTTCAGTTGAAAGACTGGACTGTTGGACTCAAGAGCTACATT<br>GCTTCATTCCTTTTTCTCTGGGACATTCAGAAATGACAACAGATGGA<br>TTCATATTGGCTGCTTGGCCAAAGAAGCGAAGAGGAATTTACTCTG<br>ATTCATTCCATTTCTTGCACAATCTCTCTCTGACTTACTAACCAATTC<br>AATTAAGCAAACCTATATTAAGCACTTACTACTTGCTCCCCACTTGC<br>AGGGTGCAGTATTAAGGTAACTACAGACACAAAGATGTGCAATAAG<br>AAGTTTCAGCCTACAAAGAGCTTGCTCCCTGGTTGAAGAGTCGGGC<br>ATATACCCATATAAAGTTATCATACGAAGGGTGAAGAGTGTGAAGAG<br>TGTTTACTTTCTTCCTGCAAAGACCTCATCTAAAGATACACACTCCA<br>TATTGTGTATTAGAAAGGAATCATTTTTGTACAAATAACTAAAAGTC<br>CACTTCAGTCTAACTAAGGGCATTAACTTACAGGAAGCCTAAGATTA<br>GATATTCAGAGAAGGCCTAGTCCAACCTAGTTTTTCTCTCACTATTT<br>CTTGGTTTATGTCCTCAGCTTATATTCATGCATGGGCAGTCTCTTTCG<br>TTTGTGTTCTAAGATGATTACCAGTGTCTTCCAGAGCTTTGTGCTTC<br>TTTATTCATATGTAACAGGAATGACAGATTGAATTTCCTGGTAGCTC<br>AAATGCAGCTCCCTCAAATCAGCCTCAGTGGTCATGGGTGGCCTGA<br>TTTGGATCATGAATATGTCTTCAAACCAGG

Supplementary Figures and Figure legends:

Figure S1

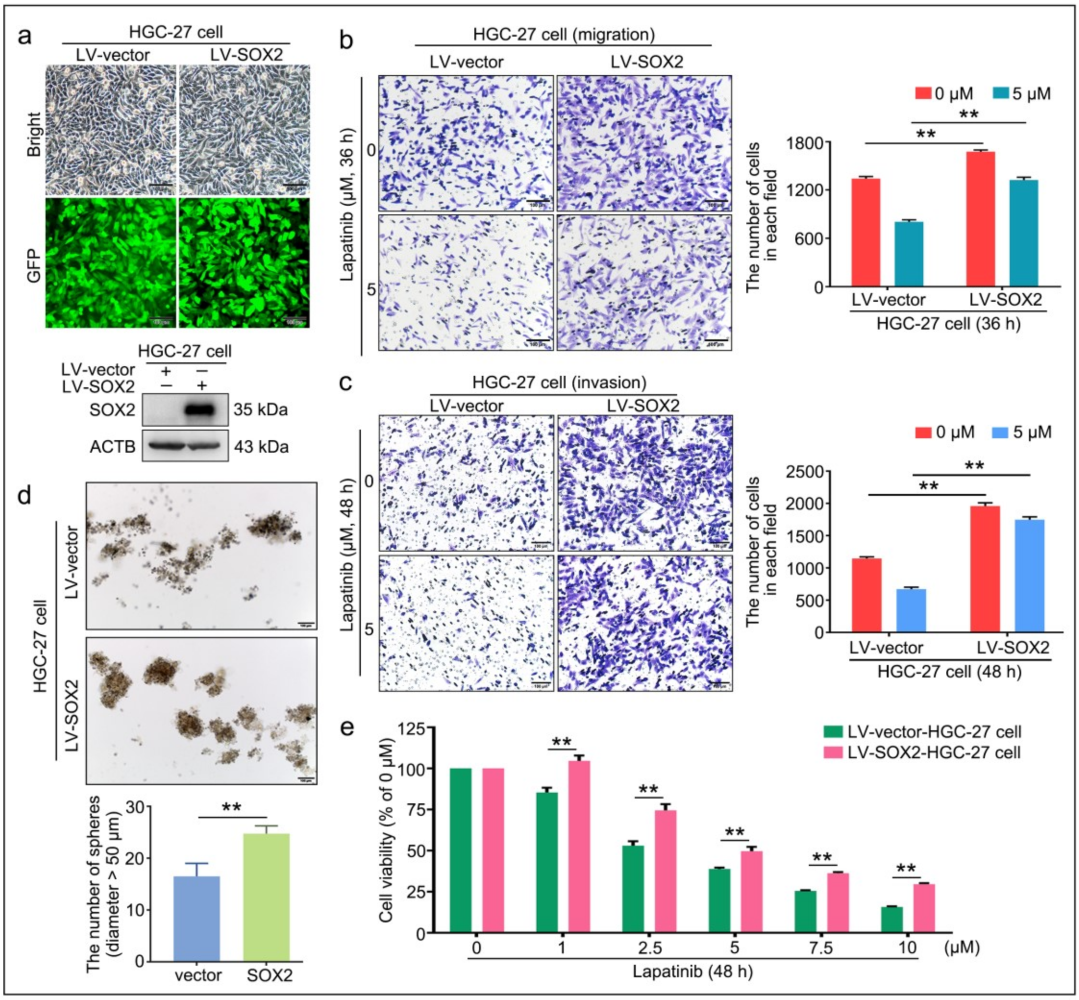

Figure S1. The overexpression of SOX2 promoted migration, invasion, self-renewal, and lapatinib resistance in HGC-27 cells. (a) Fluorescence intensity of the GFP tag. The overexpression efficiency of SOX2 in HGC-27 cells was detected by western blot. (b-c) LV-vector-HGC-27 and LV-SOX2-HGC-27 cells were treated with 5 μM lapatinib for 36 h or 48 h. Transwell assays were used to detect cell migration and invasion. Scale bar: 100 μm. (d) Sphere formation assay of LV-vector-HGC-27 and LV-SOX2-HGC-27 cells was performed after being cultured in 24-well ultra-low adherent culture plates for 4 days. The number of spheres (diameter > 50 μm) was counted. (e) LV-vector-HGC-27 and LV-SOX2-HGC-27 cells were treated with lapatinib (0, 1, 2.5, 5, 7.5, 10 μM) for 72 h. Cell viability was analyzed by CCK-8 assay. Results are mean ± SEM (**$p < 0.01$).

Figure S2

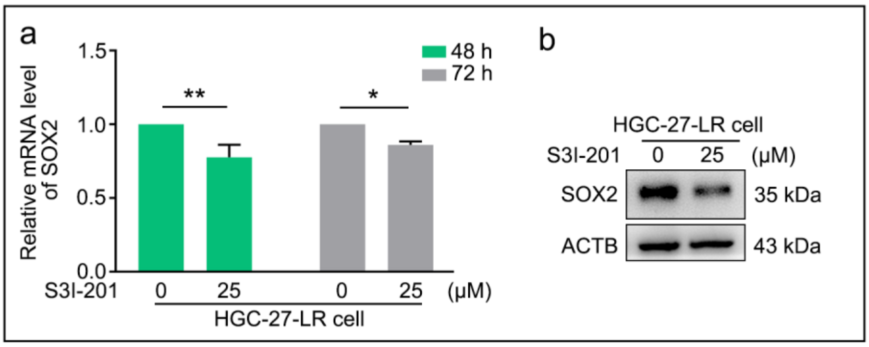

Figure S2. S3I-201 decreased the expression of SOX2 by inhibiting the DNA-binding activity of STAT3. (a) In HGC-27-LR cells, the mRNA level of SOX2 were detected by using qPCR after treatment with S3I-201 for 48 h and 72 h. (b) The protein level of SOX2 were detected by using western blot after treatment In HGC-27-LR cells with S3I-201 for 72 h. (* $p < 0.05$, ** $p < 0.01$).

Figure S3

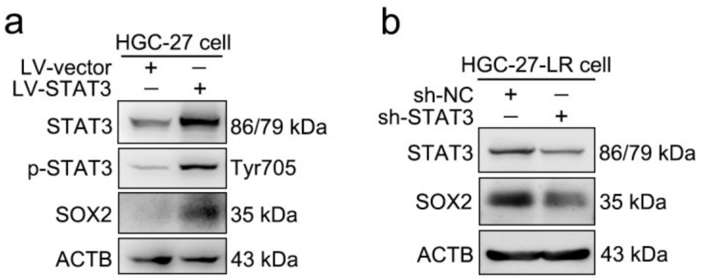

Figure S3. STAT3 positively regulates the expression of SOX2. (A) Western blot analysis of p-STAT3, STAT3, and SOX2 in LV-vector-HGC-27 and LV-STAT3-HGC-27 cells. (B) Western blot and qPCR analysis of STAT3 and SOX2 in sh-NC and sh-STAT3-HGC-27-LR cells.

Figure S4

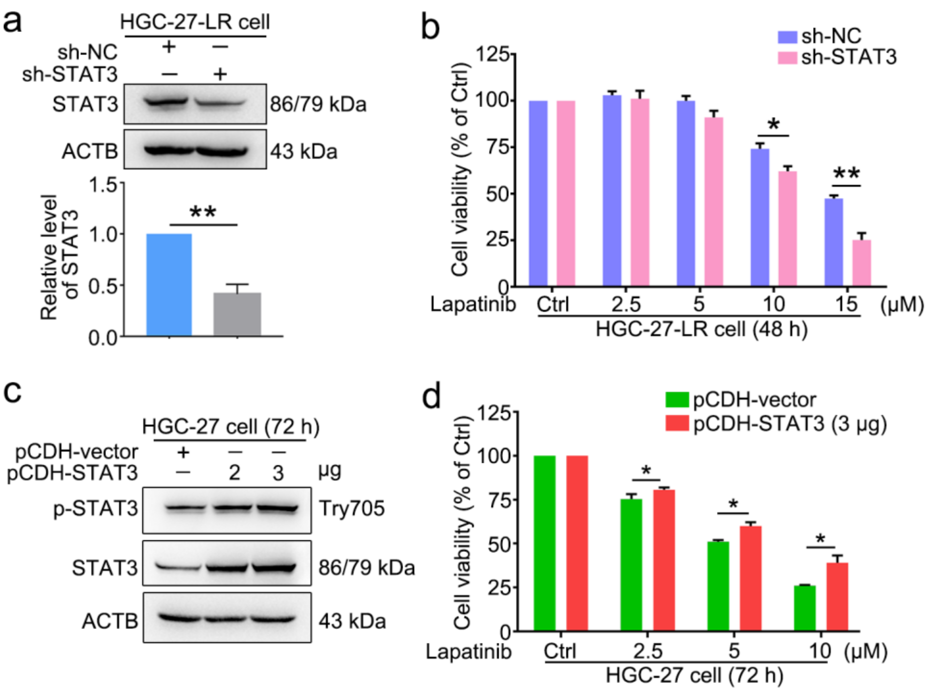

Figure S4. STAT3 promoted tolerance to lapatinib in HGC-27 cells. (A) Western blot analysis of p-STAT3 and STAT3 in sh-NC and sh-STAT3-HGC-27-LR cells. The gray values of the bands were analyzed, n = 3. (B) After treatment of sh-NC and sh-STAT3-HGC-27-LR cells with lapatinib (2.5, 5, 10, 15 μM) for 48 h, cell viability was analyzed by CCK-8. Ctrl: 0.1% DMSO (v/v). (C) HGC-27 cells were transfected with pCDH (3 μg) or pCDH-STAT3 (2 μg, 3 μg) for 72 h, and then STAT3 and p-STAT3 levels were analyzed by western blot. (D) After transfection with pCDH (3 μg) or pCDH-STAT3 (3 μg) overnight, HGC-27 cells were treated with lapatinib (2.5, 5, 10 μM) for 72 h. CCK-8 analysis of cell viability. Ctrl: 0.1% DMSO (v/v). Results are mean ± SEM (*$p < 0.05$, **$p < 0.01$).

Original images of western blots:

Statement:
All the blots were cut prior to hybridisation with antibodies.

Figure 1g

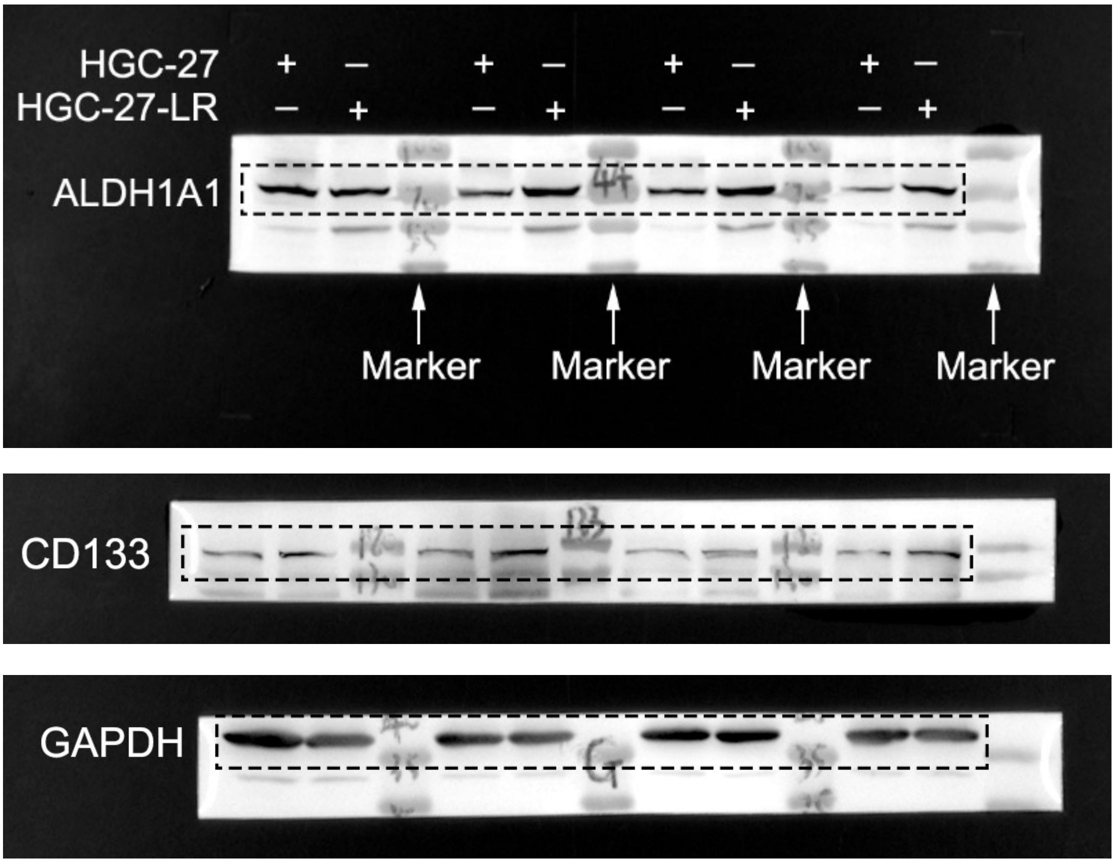

Figure 4d

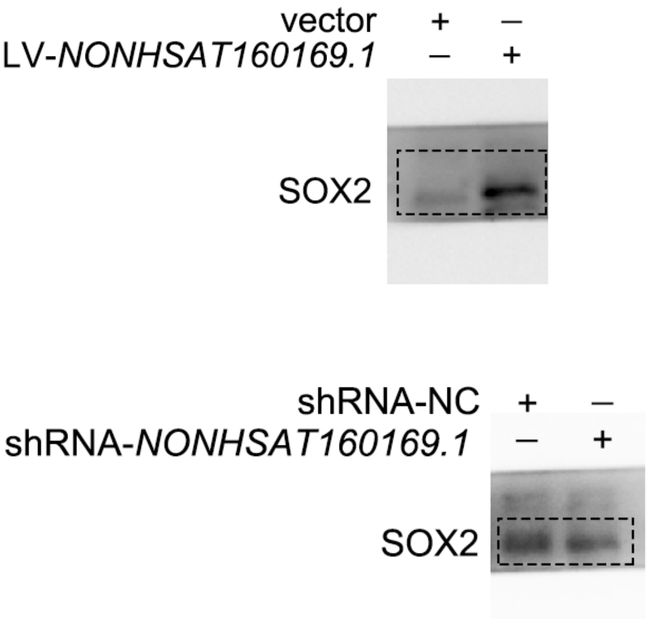

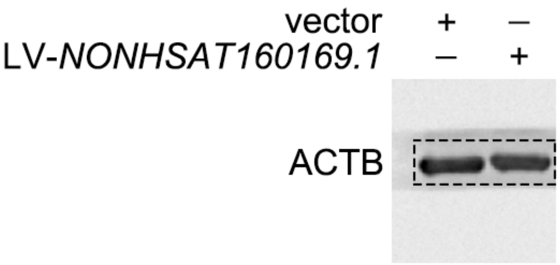
vector + —
LV-*NONHSAT160169.1* — +
ACTB

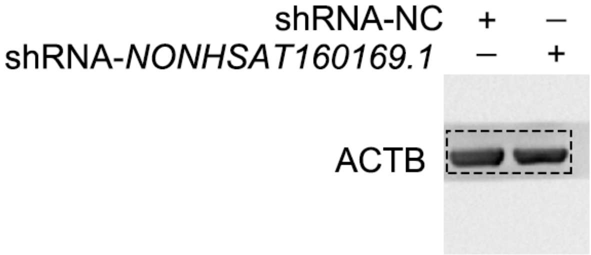
shRNA-NC + —
shRNA-*NONHSAT160169.1* — +
ACTB

Figure 4f

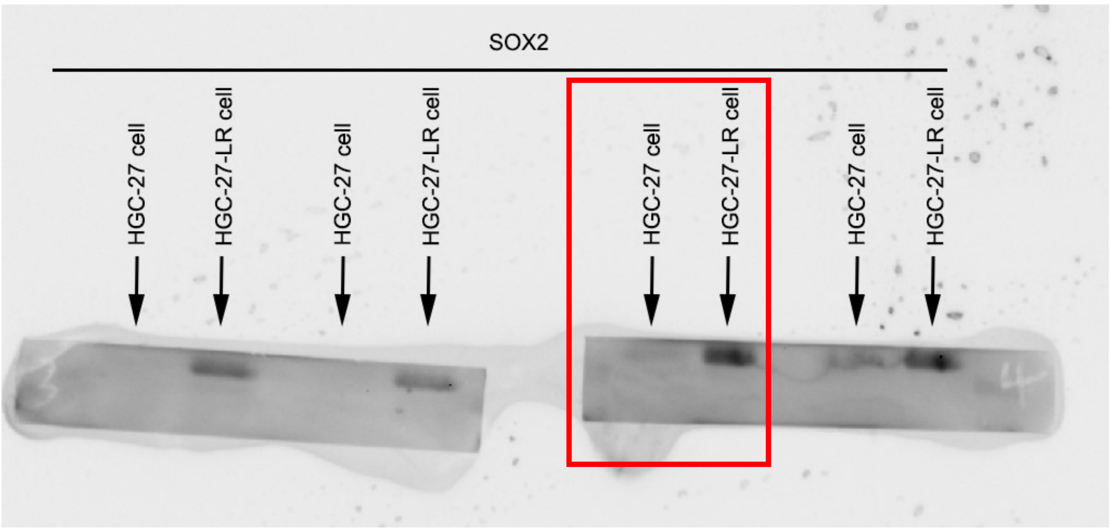
SOX2
HGC-27 cell
HGC-27-LR cell
HGC-27 cell
HGC-27-LR cell
HGC-27 cell
HGC-27-LR cell
HGC-27 cell
HGC-27-LR cell

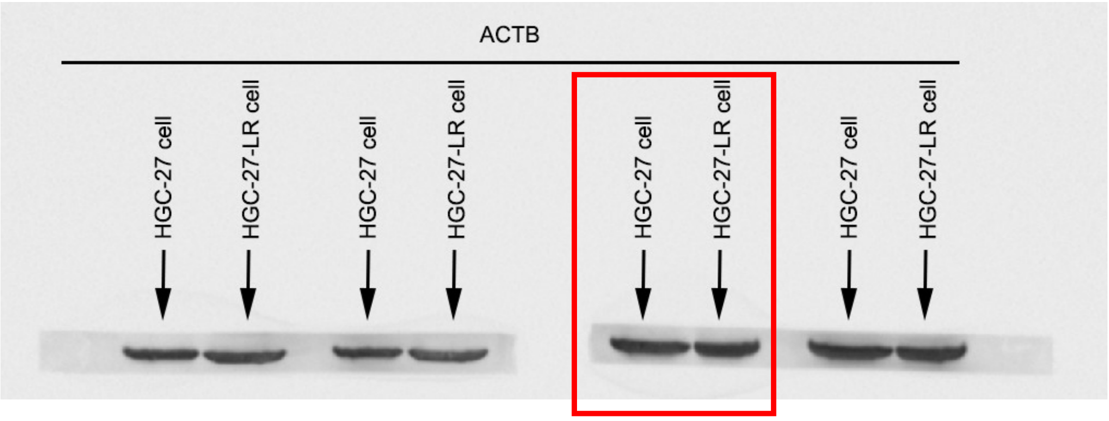
ACTB
HGC-27 cell
HGC-27-LR cell
HGC-27 cell
HGC-27-LR cell
HGC-27 cell
HGC-27-LR cell
HGC-27 cell
HGC-27-LR cell

Figure 5g

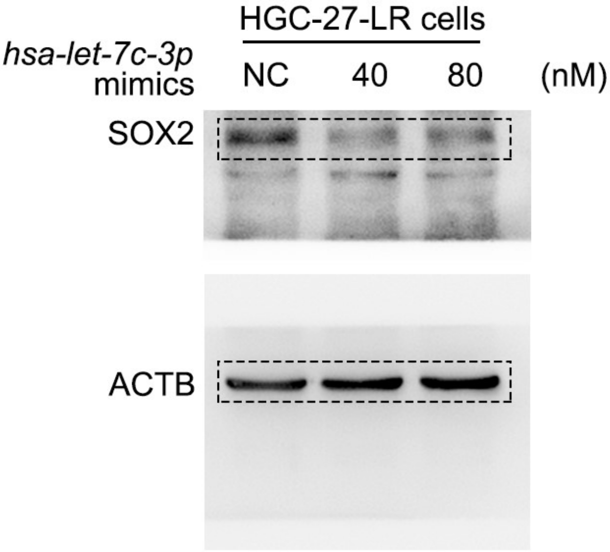

Figure 5h

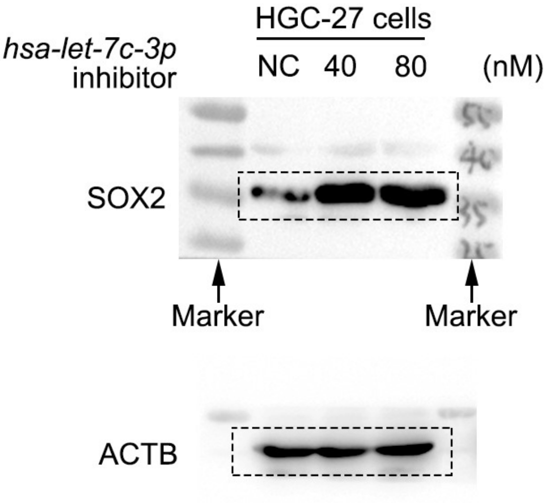

Figure 6b

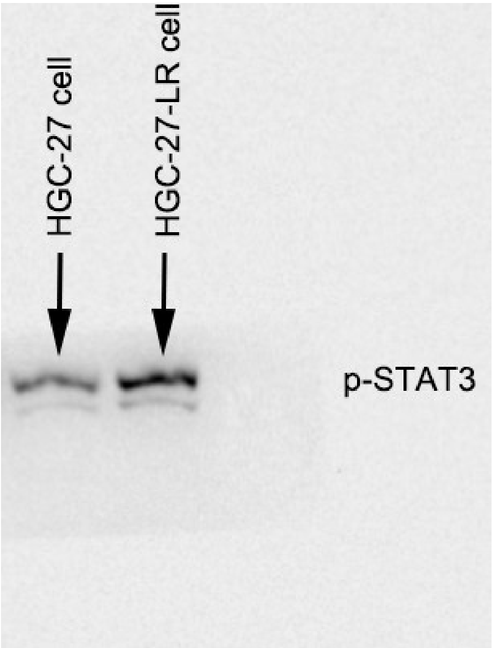
HGC-27 cell
HGC-27-LR cell
p-STAT3

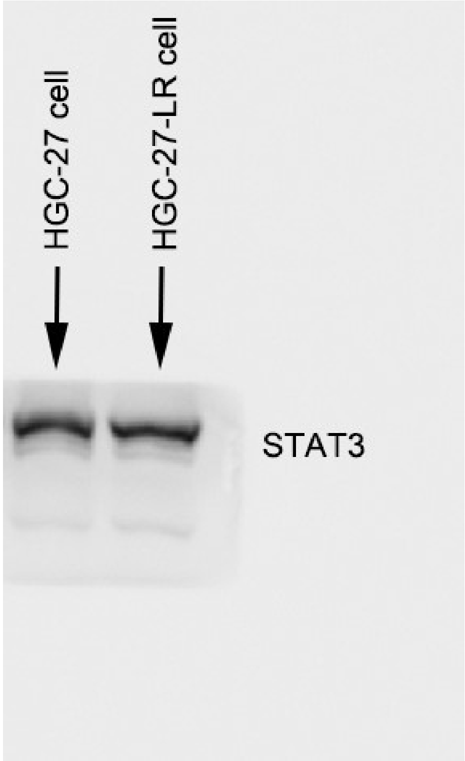
HGC-27 cell
HGC-27-LR cell
STAT3

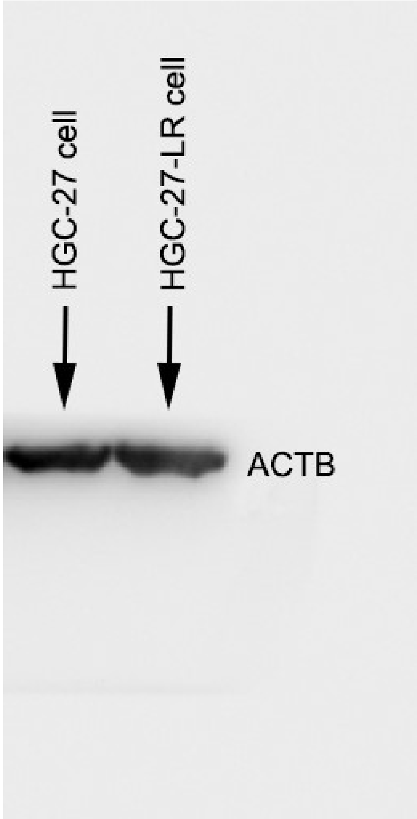
HGC-27 cell
HGC-27-LR cell
ACTB

Figure 6c

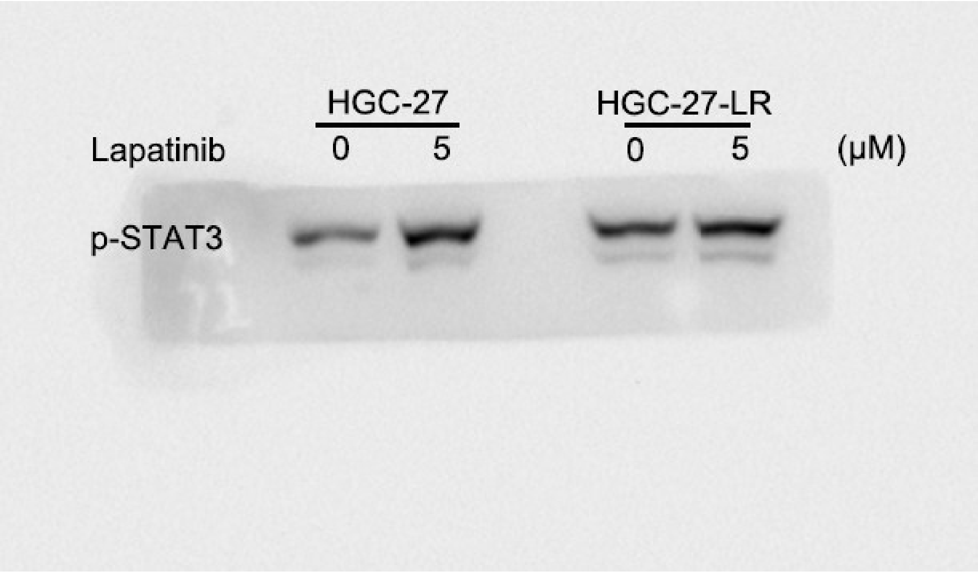
HGC-27
HGC-27-LR
Lapatinib
0
5
0
5
(μM)
p-STAT3

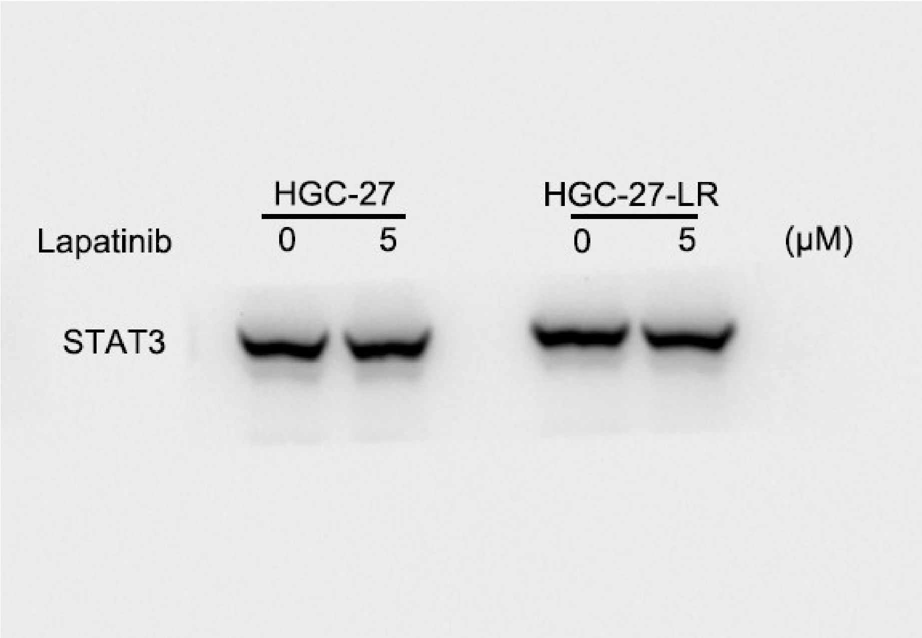
HGC-27
HGC-27-LR
Lapatinib
0
5
0
5
(μM)
STAT3

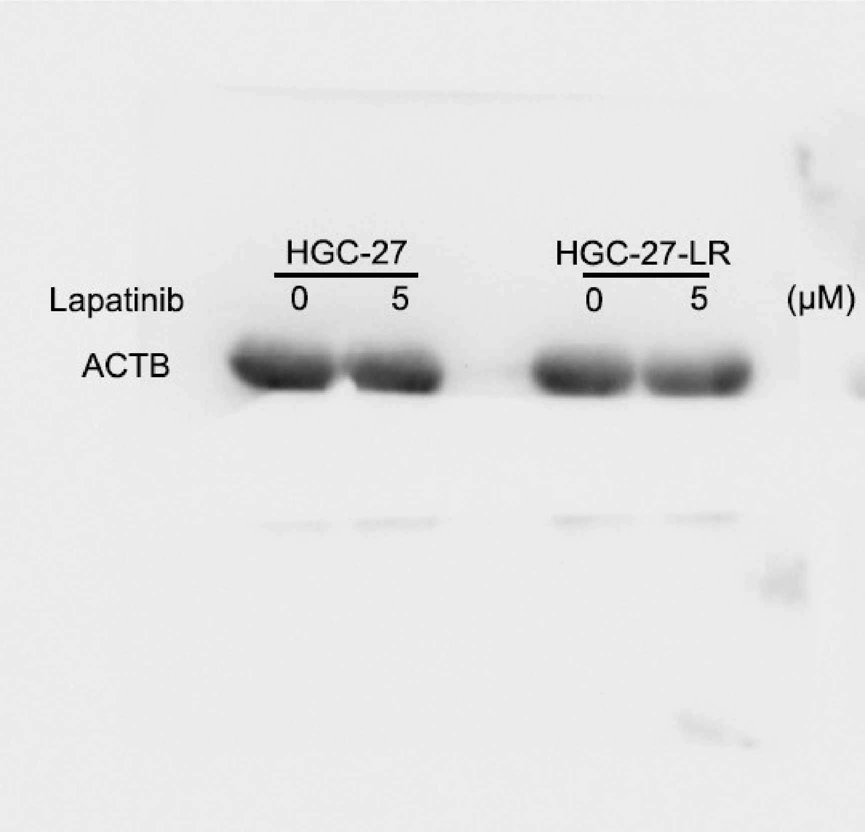
HGC-27
HGC-27-LR
Lapatinib
0
5
0
5
(μM)
ACTB

Figure 6d

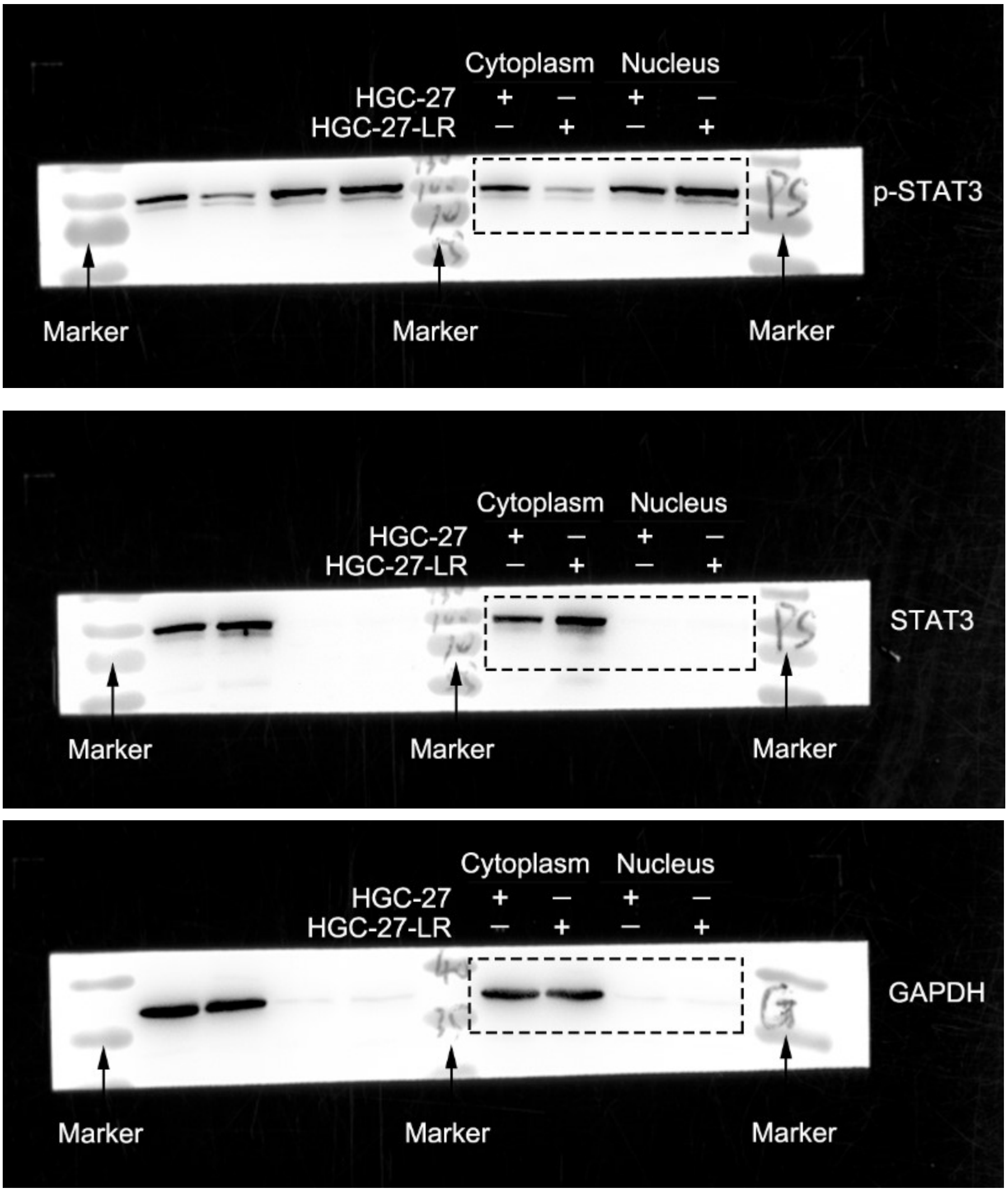
Cytoplasm
Nucleus
HGC-27 + – + –
HGC-27-LR – + – +
p-STAT3
Marker
Marker
Marker
Cytoplasm
Nucleus
HGC-27 + – + –
HGC-27-LR – + – +
STAT3
Marker
Marker
Marker
Cytoplasm
Nucleus
HGC-27 + – + –
HGC-27-LR – + – +
GAPDH
Marker
Marker
Marker

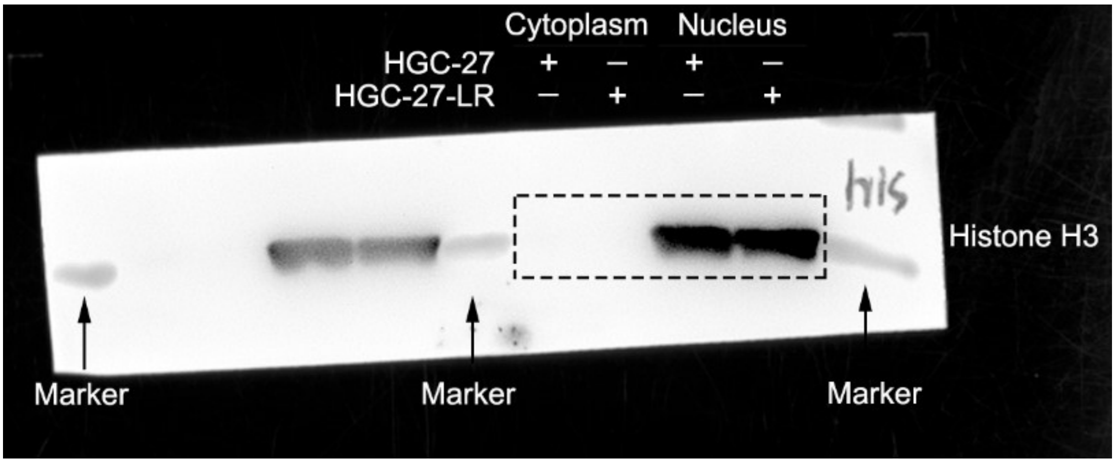

Figure S1a (Note: the protein strips in the dotted box were shown in the figure.)

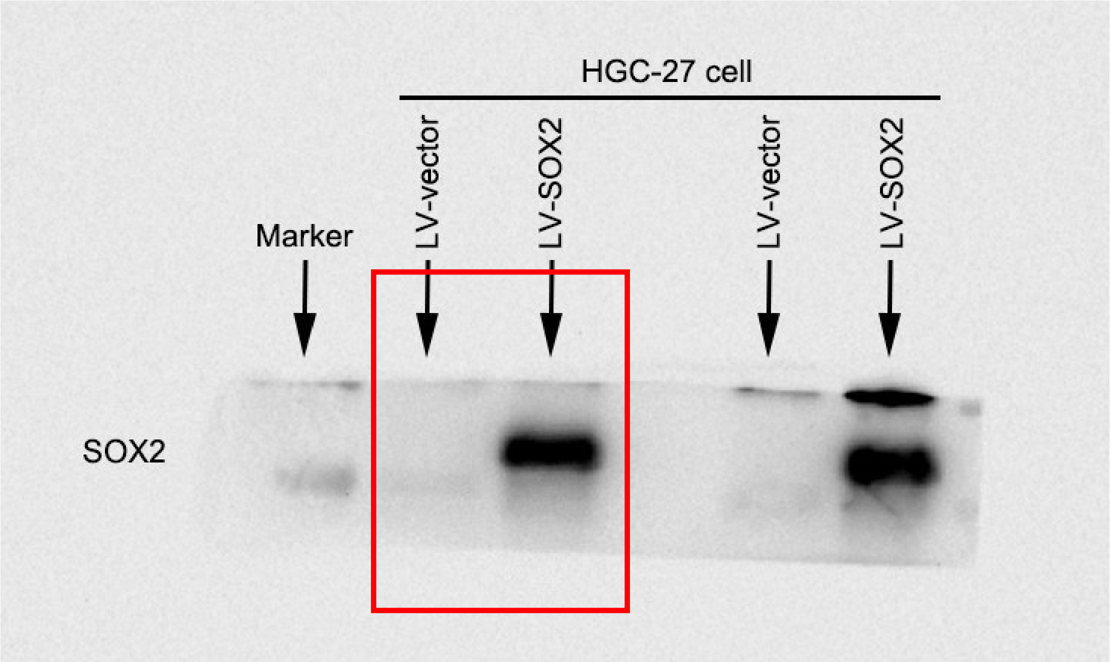

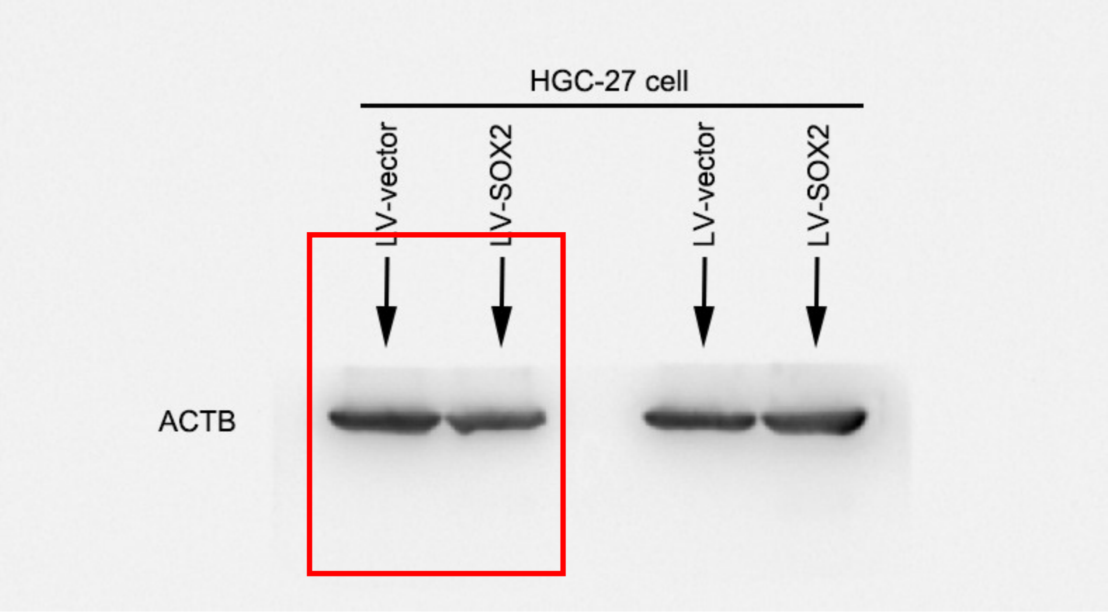

Figure S2b

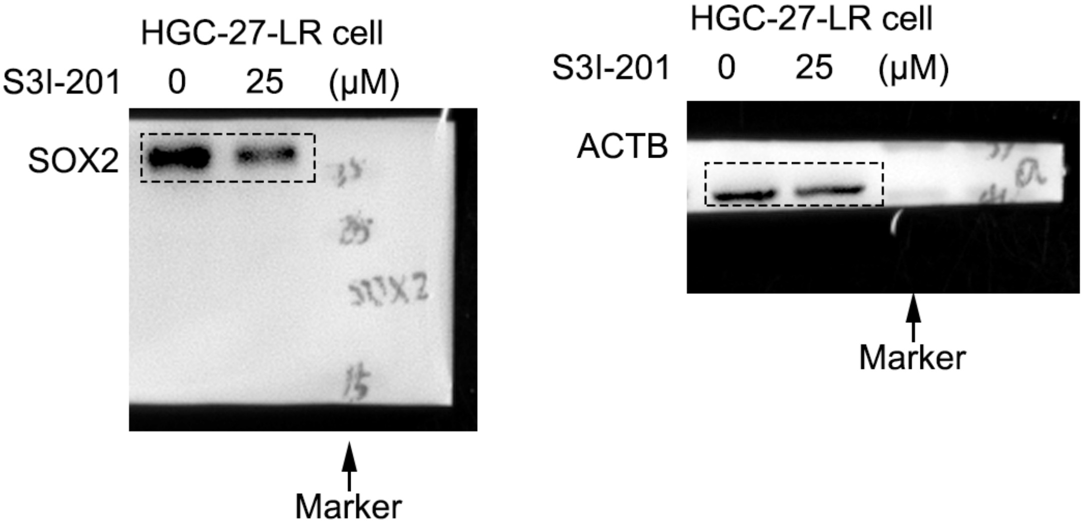

Figure S3a

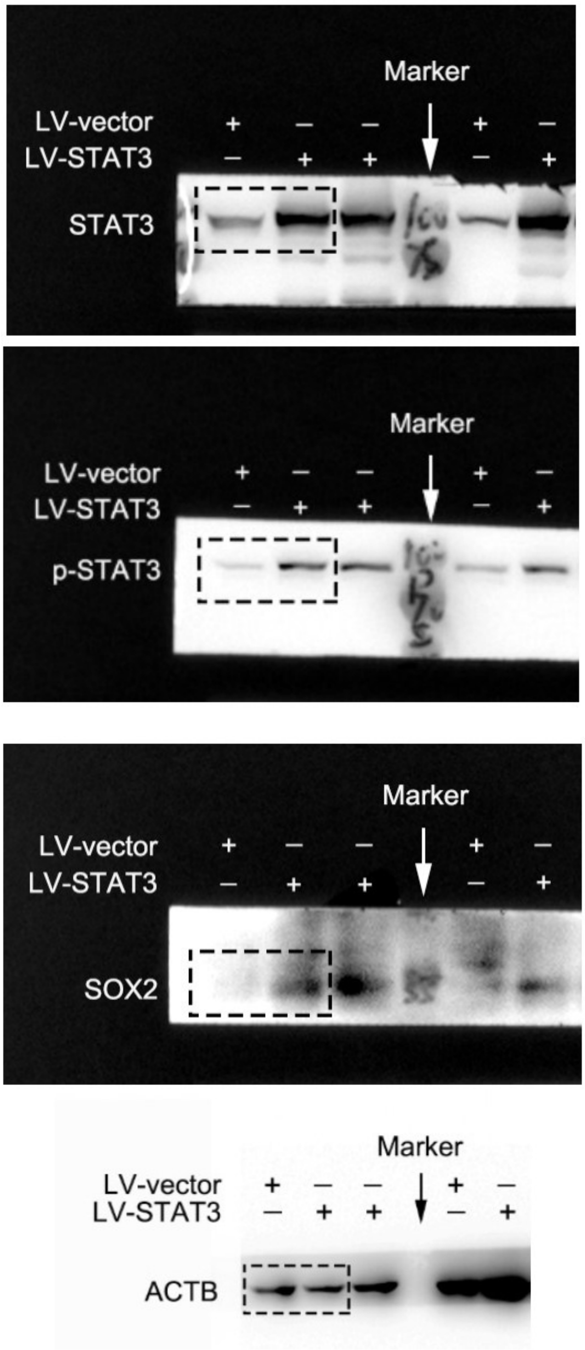
Marker
LV-vector + – – + –
LV-STAT3 – + + – +
STAT3
p-STAT3
SOX2
ACTB

Figure S3b

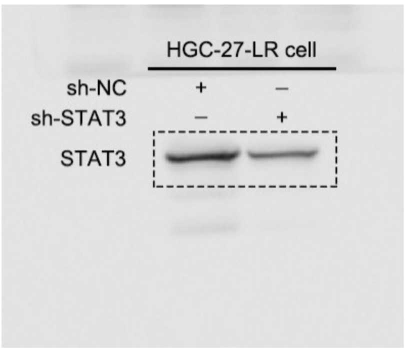
HGC-27-LR cell
sh-NC + –
sh-STAT3 – +
STAT3

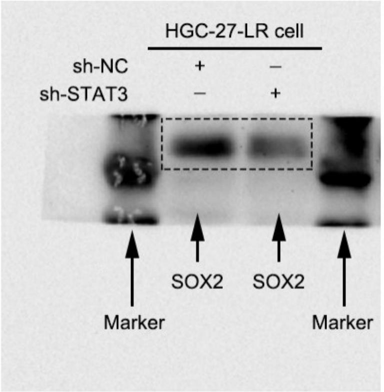
HGC-27-LR cell
sh-NC + –
sh-STAT3 – +
SOX2
SOX2
Marker
Marker

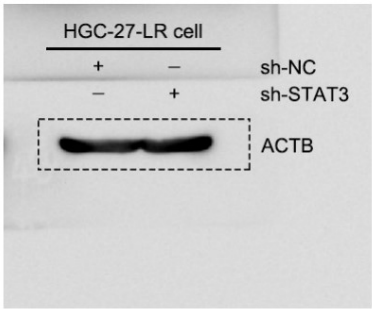
HGC-27-LR cell
+ – sh-NC
– + sh-STAT3
ACTB

Figure S4a

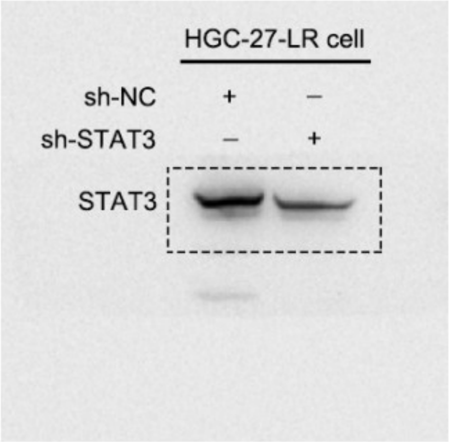
HGC-27-LR cell
sh-NC + –
sh-STAT3 – +
STAT3

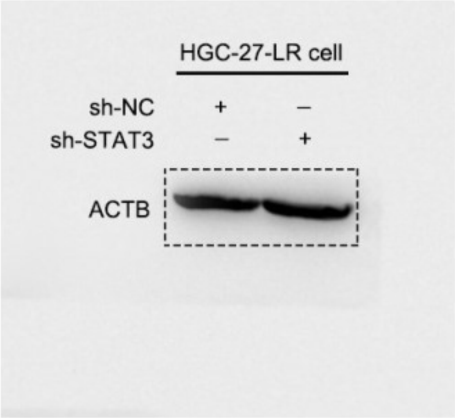
HGC-27-LR cell
sh-NC + –
sh-STAT3 – +
ACTB

Figure S4c

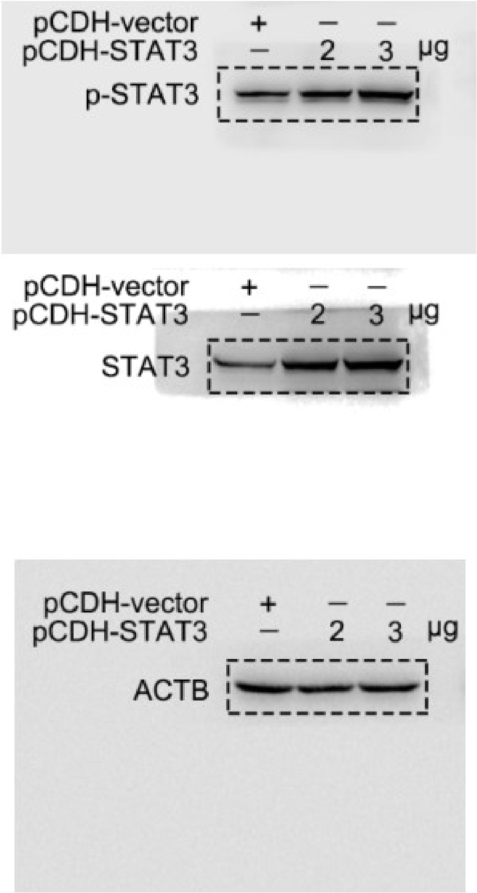
pCDH-vector + – –
pCDH-STAT3 – 2 3 μg
p-STAT3
pCDH-vector + – –
pCDH-STAT3 – 2 3 μg
STAT3
pCDH-vector + – –
pCDH-STAT3 – 2 3 μg
ACTB
